# A Facile and Novel Approach to Manufacture Paclitaxel-Loaded Proliposome Tablet Formulations of Micro or Nano Vesicles for Nebulization

**DOI:** 10.1007/s11095-020-02840-w

**Published:** 2020-06-02

**Authors:** Iftikhar Khan, Katie Lau, Ruba Bnyan, Chahinez Houacine, Matthew Roberts, Abdullah Isreb, Abdelbary Elhissi, Sakib Yousaf

**Affiliations:** 1 School of Pharmacy and Biomolecular Sciences, Liverpool John Moores University, Liverpool, L3 3AF UK; 2 School of Pharmacy and Biomedical Sciences, University of Central Lancashire, Preston, PR1 2HE UK; 3 Pharmaceutical Sciences Section, College of Pharmacy, Qatar University, P.O. Box 2713, Doha, Qatar

**Keywords:** nebulizer, paclitaxel, proliposome, quality control testing, tablets

## Abstract

**Purpose:**

The aim of this study was to develop novel paclitaxel-loaded proliposome tablet formulations for pulmonary drug delivery.

**Method:**

Proliposome powder formulations (i.e. F1 – F27) were prepared employing Lactose monohydrate (LMH), Microcrystalline cellulose (MCC) or Starch as a carbohydrate carriers and Soya phosphatidylcholine (SPC), Hydrogenated soya phosphatidylcholine (HSPC) or Dimyristoly phosphatidylcholine (DMPC) as a phospholipid. Proliposome powder formulations were prepared in 1:5, 1:15 or 1:25 *w*/w lipid phase to carrier ratio (lipid phase; comprising of phospholipid and cholesterol in 1:1 M ratio) and Paclitaxel (PTX) was used as model anticancer drug.

**Results:**

Based on flowability studies, out of 27 formulations; F3, F6, and F9 formulations were selected as they exhibited an *excellent* angle of repose (AOR) (17.24 ± 0.43, 16.41 ± 0.52 and 15.16 ± 0.72°), comparatively lower size of vesicles (i.e. 5.35 ± 0.76, 6.27 ± 0.59 and 5.43 ± 0.68 μm) and *good* compressibility index (14.81 ± 0.36, 15.01 ± 0.35 and 14.56 ± 0.14) via Carr’s index. The selected formulations were reduced into Nano (N) vesicles via probe sonication, followed by spray drying (SD) to get a dry powder of these formulations as F3SDN, F6SDN and F9SDN, and gave high yield (>53%) and exhibited *poor* to *very poor* compressibility index values via Carr’s Index. Post tablet manufacturing, F3 tablets formulation showed uniform weight uniformity (129.40 ± 3.85 mg), good crushing strength (14.08 ± 1.95 N), precise tablet thickness (2.33 ± 0.51 mm) and a short disintegration time of 14.35 ± 0.56 min, passing all quality control tests in accordance with British Pharmacopeia (BP). Upon nebulization of F3 tablets formulation, Ultrasonic nebulizer showed better nebulization time (8.75 ± 0.86 min) and high output rate (421.06 ± 7.19 mg/min) when compared to Vibrating mesh nebulizer. PTX-loaded F3 tablet formulations were identified as toxic (60% cell viability) to cancer MRC-5 SV2 cell lines while safe to normal MRC-5 cell lines.

**Conclusion:**

Overall, in this study LMH was identified as a superior carbohydrate carrier for proliposome tablet manufacturing in a 1:25 *w*/w lipid to carrier ratio for *in-vitro* nebulization via Ultrasonic nebulizer.

**Electronic supplementary material:**

The online version of this article (10.1007/s11095-020-02840-w) contains supplementary material, which is available to authorized users.

## Introduction

The usage of Paclitaxel (PTX) as an anticancer agent in the treatment of a plethora of cancers (e.g. lung, breast and neck) is well documented (1). PTX appears as needle or fine white to off-white crystalline powder with a molecular weight of 853.91 g/mol (chemical structure of PTX in supplementary data (Fig. a)). PTX storage temperature is 0°C for short term and − 20°C for long term in desiccator. A number of physicochemical properties, like; size, size distribution, entrapment efficiency and zeta potential are critical parameters for PTX-loaded formulations. PTX is associated with a number of physicochemical limitations, two significant issues include; lower aqueous solubility and cellular permeability (2,3). A number of approaches have been employed to overcome these drawbacks, including the use of cyclodextrins, which significantly improve PTX water solubility without altering its cytostatic activity (4). A commercially available parenteral formulation under the name of Taxol® is available on the market, which has addressed the compounds poor solubility by formulation with poly-oxyethylated castor oil and ethanol in a 50:50 *v*/v ratio followed by 5–20 times dilution. However, there are established side-effects associated with poly-oxyethylated castor oil, namely, laboured breathing and hypersensitivity reactions (5). Thus, there is a clear need for a biocompatible formulation, which enhances cellular permeability and water solubility. Previous research has addressed this need through formulation of PTX loaded onto albumin nanoparticles, under the product Abraxane® (currently under clinical trial). Whilst this has been observed to improve drug permeation (circa 33%) when compared to Taxol®, human albumin itself is of high monetary value, raising questions surrounding affordability. An alternative medium or carrier system is hence desirable to encapsulate PTX to aid delivery (6). In comparison to human albumin, lipids are cheap and provided possible use in animal model. Using these lipids, liposomes can be manufactured on large scale using fluid-bed technology in conjugation with freeze drying (7). The average half-life of PTX followed by intravenous administration is reported to be between 3 and 50 h (8,9). However, pulmonary administration of PTX has been reported to enhance drug accumulation within the lung and reduce systemic distribution, which in turn exhibited reduced systemic toxicity (10). Similarly, an *in-vitro* study demonstrated complete tumour remission and reduced systemic toxicity post nebulization of PTX delivered using solid lipid nanoparticles (11). Likewise, transfersome/protransfersome is an another lipid-based drug delivery system designed to suit different situations and delivery scenarios, however unlike liposome and solid lipid nanoparticles incorporate a surfactant as the primary component, imbibing transfersome with elasticity in terms of their structure enhancing delivery performance, also demonstrated cytotoxic activity against cancer cells (12). A comparison between PTX liposome formulation as aerosol and the intravenous administration of two commercial formulations was conducted by Joshi, Shirsath (13). The study demonstrated no pulmonary toxicity following aerosol treatment with 75% more enhanced metastases inhibition, in comparison to parenteral administration (13).

The biocompatibility and biodegradability of liposomes (lipid vesicles) are well-established properties (14). Being structurally similar to biological membranes, unlike poly-oxyethylated castor oil, are not associated with hypersensitivity reactions (15,16). Hydrophobic compounds are commonly entrapped within the concentric bilayers of the vesicles, whereas hydrophilic molecules position into the central aqueous core (17). A number of studies have demonstrated that PTX can be entrapped into liposomes bilayers without PEGylation (18–22). Additionally, PTX entrapment and stability in bilayers may be affected by a number of factors (e.g. presence of saturated and unsaturated hydrocarbon chains of lipids, PEGylation, fusion or aggregation, and the presence or absence of triglycerides (23)). PEGylation coating of liposomes may also prolong retention time, reduce recognition and uptake by phagocytic cells, offer protection from opsonin binding in plasma (24–26), thus improving pharmacokinetic and pharmacodynamics properties (27) (i.e. long circulating liposomes) (28). As formulations, drug-loaded liposomes delivered to the lungs via nebulization have been associated with a lower incidence of system side-effects and improved therapeutic effect (29). Arikace® is a liposome formulation containing amikacin for the treatment of cystic fibrosis via nebulization is available on the market. The success of this formulation may enhance the use of liposomal formulation for various lung diseases (30).

However, a notable limitation of liposomes however is poor stability upon storage (31–33) exhibited by aggregation or fusion of vesicles and drug leakage from the vesicles (17,34). The formulation of liposomes in a dry-stable form referred to as proliposomes (which may be hydrated with water to generate liposomes) has been developed as a solution to stability issues associated with the vesicles (35,36). Succinctly, proliposomes are formed from a combination of drug and phospholipid (with or without cholesterol) termed the lipid component, which is uniformly distributed over carbohydrate carrier particles (14,37,38). It is important to recognize that liposomes generated from proliposomes (via hydration using water as aqueous medium) are typically micrometre in size, with particles sized larger than 5 μm generally depositing in the upper respiratory tract (33,39) (i.e. from mouth to trachea), hence retarding drug deposition in the lower respiratory area (from bronchi to bronchioles) of pulmonary system for its pharmacological action. Therefore, size reduction is needed in order to reduce particle size from micrometre into nanometre range, in order to improve and enhance drug deposition in the middle to lower respiratory tract.

Whilst powder formulations enhance stability, they are associated with challenges due to their bulky nature, and risk associated inhalation of small solid particles during large scale manufacture (40). This may be remedied simply by formulation as tablet, which retain stability yet offer economic feasibility and convenience in handling and packaging (41). Widely manufactured in the pharmaceutical industry, in addition to an active pharmaceutical Ingredient tablets are formulated with excipients, including diluents/fillers to achieve bulk in the form of carbohydrates (42,43). Proliposomes hence improve stability of formulation, extending the product shelf-life significantly, and can be hydrated for aerosolization using nebulizers for effective pulmonary drug delivery. Nebulization is an effective technique for targeting the lungs, which provide a large surface area for drug deposition. Additionally, the technique offers needle free drug administration, improving patient compliance with minimal training and can be administered to unconscious patients delivering the desired drug via normal tidal breathing.

In the present study, proliposome dry formulations were prepared in a large quantity when compared to traditional liposome formulations (i.e. thin-film method) using a rotary evaporator to deposit a thin film of lipid phase over the carbohydrate particles. Furthermore, to develop novel PTX-loaded proliposome tablet from proliposome powder formulations, followed by their examination and selection of better drug delivery carrier to the pulmonary system using nebulizers.

## Materials and Methods

### Materials

Soya Phosphatidylcholine (SPC; Lipoid S-100; 94% purity), Hydrogenated Soya Phosphatidylcholine (HSPC; Phospholipon, 80% purity) and Dimyristoly Phosphatidylcholine (DMPC; Lipoid-PC, 98% purity) were purchased from Lipoid, Steinhausen, Switzerland. Paclitaxel (PTX) was obtained from ChemieTek, Indianapolis, USA). Analar grade methanol, acetonitrile, absolute ethanol deionized water (DW) and milipore filter (10 kDa) were purchased from Fisher Scientific Ltd., UK. Cholesterol and Fetal bovine serum (FBS) was supplied by Sigma-Aldrich, UK. Lactose Monohydrate (LMH), Microcrystalline Cellulose (MCC; Avicel PH 12), and Starch (Starch-150) were purchased from VWR (BDH Prolab), UK. The Vibrating mesh (Omron NE U22) nebulizer was supplied by Omron Healthcare UK Ltd., UK. The Ultrasonic nebulizer was purchased from Uniclife Healthcare LTD, UK. Dulbecco’s Modified Eagle Medium (DMEM) with high glucose, TryplTrypLE™ Express Enzyme (1X), with phenol red, and AlamarBlue™ cell viability reagent were bought from Gibco, Thermofisher, UK. MRC-5 (ECACC 05090501) and MRC-5 SV2 (ECACC 84100401) cell lines were obtained from Public Health England (Salisbury, UK).

### Preparation of PTX-Loaded Proliposome Formulation

Proliposome powders were prepared via slurry method (17,36). Combinations of one of three different phospholipids i.e. SPC, HSPC or DMPC and one of three different carbohydrate carriers i.e. LMH, MCC or Starch were employed in proliposome formulations. SPC and HSPC are from natural source, whereas DMPC is from synthetic source. The lipid phase (250 mg) prepared, consisted of phospholipid and cholesterol in 1:1 M ratio. Three different formulation ratios of lipid phase to carrier were prepared i.e. 1:5, 1:15 and 1:25 (*w*/w). A model drug PTX was incorporated at a 2 mol% concentration based on lipid phase. In formulation of 1:5 w/w lipid to carrier ratio proliposomes, 1250 mg of LMH was transferred to a round bottom flask (RBF) (100 ml). A lipid phase, comprised of SPC (166.66 mg) and cholesterol (83.33 mg) with 2 mol% of PTX (7.34 mg) was dissolved in 20 ml of absolute ethanol; this solution was then poured over the LMH forming a slurry. Subsequently, the RBF was subjected to rotary evaporation (Buchi Rotavapor R-114, Buchi, Switzerland) in a water bath (Buchi Water bathe B-480, Buchi, Switzerland) previously adjusted to 45°C. A negative pressure was created by a vacuum pump (Buchi Vac V-501) and the evaporation of organic solvent was continued with a rotation speed of 270 RPM for 1 h. Following release of negative pressure, dry proliposome (i.e. powder form) were collected in a dry air-tight glass bottle (100 ml) and stored at −18°C for subsequent studies. This procedure was repeated using various phospholipids, carbohydrate carriers and lipid phase to carrier ratios to prepare 27 proliposome powder formulations (Table I).

**Table I:** Proliposome Formulations (F1 – F27) Prepared Employing Three Different Phospholipids (i.e. SPC, HSPC and DMPC), Three Different Carbohydrate Carriers (i.e. LMH, MCC and Starch) and Three Different Lipid Phase to Carrier Ratios (i.e. 1:5, 1:15 and 1:25 *w*/w). Lipid Phase was Prepared by Using Phospholipid and Cholesterol in a 1:1 M ratio, *n* = 3

Formulation name	Carbohydrate carrier	Phospholipid	Lipid phase to carrier ratio (w/w)
F1	LMH	SPC	1:5
F2	LMH	SPC	1:15
F3	LMH	SPC	1:25
F4	LMH	HSPC	1:5
F5	LMH	HSPC	1:15
F6	LMH	HSPC	1:25
F7	LMH	DMPC	1:5
F8	LMH	DMPC	1:15
F9	LMH	DMPC	1:25
F10	MCC	SPC	1:5
F11	MCC	SPC	1:15
F12	MCC	SPC	1:25
F13	MCC	HSPC	1:5
F14	MCC	HSPC	1:15
F15	MCC	HSPC	1:25
F16	MCC	DMPC	1:5
F17	MCC	DMPC	1:15
F18	MCC	DMPC	1:25
F19	Starch	SPC	1:5
F20	Starch	SPC	1:15
F21	Starch	SPC	1:25
F22	Starch	HSPC	1:5
F23	Starch	HSPC	1:15
F24	Starch	HSPC	1:25
F25	Starch	DMPC	1:5
F26	Starch	DMPC	1:15
F27	Starch	DMPC	1:25

### Carr’s Consolidation Index and Angle of Repose

For Carr’s consolidation index, a graduated cylinder (25 ml) was employed (44,45). For each coarse carbohydrate carrier, 10 g of the measured powder was transferred to a cylinder followed by gentle tapping (2–3 repetitions) in order to level the powder in the cylinder. The initial volume (V_1_) of the powder was recorded; subsequently, the cylinder was affixed to an automated controlled tapping tapped density tester (Agilent technologies, USA). The height of the tapped cylinder was set at 14 ± 2 mm, with a tapping rate of 100 ± 15 tapes/min for 5 min. The final volume (V_2_) of powder in the cylinder was recorded after 5 min period. Carr’s consolidation Index was calculated via Eq. 1 using the values generated from this experiment. The same experiment was repeated for all coarse carbohydrate carriers (i.e. LMH, MCC and Starch) in addition to prepared proliposome powder formulations (i.e. F1 – F27) (Table I).

1 $$\mathrm{Car}{\mathrm{r}}^{\prime}\mathrm{s}\ \mathrm{Index}\ \left(\%\right)=\left(\frac{\ \mathrm{V}2-\mathrm{V}1}{\mathrm{V}2}\right)\times 100$$

In measuring angle of repose (AOR), a funnel was set up on a clamp and stand, a height of circa 2–4 cm distance was adjusted from the end of the funnel to the table surface. The weight of the powder (10 g) was first recorded and fed through the funnel to form a uniform cone (46). The height of the powder cone was then recorded along with the diameter of the powder base. In the event of improper cone formation, the experiment was repeated to achieve uniform symmetrical cone formation and subsequent accurate measurement. AOR was calculated for three carbohydrate carriers (i.e. LMH, MCC and Starch) and proliposome powder formulations (F1 – F27) with the help of following Eq. 2;

2 $$\mathrm{Angle}\ \mathrm{of}\ \mathrm{Repose}={\tan}^{-1}\left(\frac{\mathrm{Powder}\ \mathrm{height}\ \left(\mathrm{cm}\right)}{\mathrm{Powder}\ \mathrm{base}\ \left(\mathrm{cm}\right)}\right)$$

The degree of powder flow in accordance with Carr’s scale for compressibility index and AOR are exhibited in order to show powder characteristics (Table II) (47).

**Table II:** Representing Flow Properties of Powder Using Compressibility Index and Angle of Repose Via Carr’s Scale of Flowability (47)

Compressibility index (%)	Flow character	Angle of repose (degree)
1–10	Excellent	25–30
11–15	Good	31–35
16–20	Fair	36–40
21–25	Passable	41–45
26–31	Poor	46–55
32–37	Very poor	56–65
˃ 38	Very, very poor	˃ 65

### Size and Zeta Potential Analysis

Liposomes were generated from proliposome powder formulations via hydration using water as a dispersion medium (30 mg/ml) followed by 1 h annealing time (i.e. conducted above the phase transition temperature of phospholipid). The phase transition temperature of SPC, HSPC and DMPC are −20, 52, and 23°C, respectively. Liposome vesicle size (also referred to as Volume Median Diameter; VMD) and PDI (referred to as size distribution) analysis of liposome generated from proliposomes were conducted using laser diffraction (Malvern Mastersizer 200, Malvern Instruments, UK).

Liposome vesicle size and PDI in nanometre range was measured via Dynamic Light Scattering (DLS) instrument (Zetasizer Nano; Malvern Instruments Ltd., UK). Zeta potential of liposome suspension was determined by Zetasizer Nanoseries (Malvern Instruments Ltd., UK) using Laser Doppler Velocimetry (LDV) via electrophoretic mobility of liposomes in dispersion medium. Zeta potential is the term used to define the potential difference between the conducting liquid and liposome vesicles immersed in the liquid dispersion (48). Zeta potential is the overall charge liposome vesicle acquires in a dispersion medium and it is calculated by determining the velocity of liposome vesicles in an electric field, also termed as electrophoretic mobility of the vesicles. Therefore, Zetasizer was employed to measure Zeta potential of both Micro and Nano particles.

### Entrapment Efficiency of PTX

Entrapment of PTX incorporated into the proliposome formulations was studied using HPLC (High Performance Liquid Chromatography). Following hydration and annealing of the proliposome powders into liposome suspensions (300 mg/10 ml), 1 ml of liposome suspension was diluted with methanol to determine the total amount of drug via HPLC (Agilent 1200 HPLC instrument, UK). For the unentrapped component, 0.5 ml of liposome suspension was transferred to a Millipore filter (10 kDa) (Fischer Scientific, UK) and centrifuged (Spectrafuge 24D, Labnet International, USA) at 15,100 g for 30 min. This allowed for the separation of the PTX-loaded liposomes from the unentrapped portion of PTX in suspension. The filtrate (i.e. unentrapped PTX) was diluted with methanol and the entrapped PTX was calculated in accordance with Eq. 3. The appropriate Millipore filter was selected on the basis of molecular weight of the target solute (i.e. PTX = 853.91 g/mol, which is 5–10 times smaller than the pores of Millipore filter 10 kDa), thus enabled PTX free molecules to pass through Millipore filter as filtrate, whilst retaining liposome vesicles in the Millipore filter due to their large size (which are collectively a very large assembly of countless number of phospholipid and cholesterol molecules arranged in the lamellar phase (with entrapped paclitaxel being associated with this assembly)). These differences between paclitaxel and liposomes permitted the unentrapped paclitaxel to pass preferentially through the filters pores whilst liposomes with the entrapped paclitaxel were retained by the filter.

3 $$\mathrm{Entrapment}\ \mathrm{Efficiency}\ \left(\%\right)=\left(\frac{\ \mathrm{Total}\ \mathrm{drug}\ \mathrm{loading}-\mathrm{Unentrapped}\ \mathrm{drug}}{\mathrm{Total}\ \mathrm{drug}\ \mathrm{loading}}\right)\times 100$$

PTX was assayed using a mobile phase of Acetonitrile and DW (deionized water) (80:20 *v*/v) with an injection volume of 5 μl and flow rate of 1.2 ml/min. The temperature was set and maintained at 40°C with a detection wavelength of 230 nm. The HPLC column used was ODS C-18 (150 mm X 4.6 mm) of 5-μm column (Agilent technology, USA). A standard calibration curve of PTX was constructed from 5 to 80 mg/ml.

### Scanning Electron Microscopy (SEM) and Transmission Electron Microscopy (TEM)

SEM was employed for surface morphology determination of proliposome powder, with TEM utilized for the liposome suspension. For SEM, proliposome sample powders were transferred to an aluminium stub. An air spray was employed to remove excess powder from the stub. The samples were subsequently coated with gold via JFC-1200 Fine Coater (JEOL, Tokyo, Japan) under vacuum for 2 min. Proliposome particles were observed under SEM (Quanta 200, Czech Republic) at 20 kV and images were captured. For TEM, following the generation of liposomes from proliposome, a drop of liposome suspension was transferred to a carbon coated copper grid (400 mesh) (TAAB Laboratories Equipment Ltd., UK). The sample was negatively stained with 1% *w*/*v* phosphotungstic acid, and then viewed and photographed using a Philips CM 120 Bio-Twin TEM (Philips Electron Optics BV, Netherlands).

### Spray Drying of Liposome Suspension Post Nano Sizing

Upon investigation of F1 – F27 formulations, few formulation were selected on the basis of consolidation index, AOR, size, Zeta potential and entrapment efficiency. Liposome suspensions (hydrated as 300 mg/10 ml) (vesicles in Micro size) were probe sonicated in to Nano size followed by spray drying (SD) to obtain a dry powders of proliposome. Probe sonication (Q125 Qsonica, USA) was employed for a total of 10 min with only 7 min of sonication time (i.e. 2 min sonication followed by 1 min break) at 80% of amplitude intensity. High intensity and longer sonication time was used due to the high viscosity of formulation caused by the high amount of dissolved carbohydrate carrier (1:25 *w*/w lipid phase to carrier ratio), which were also reported previously in literature (49,50). Probe sonication was applied because of its simplicity and rapidity at reducing the size of liposomes to the Nano range. Since titanium (produce during probe sonication) has much higher density (being in the Micro size range) than Nano liposomes, centrifugation was conducting using bench centrifuge (Spectrafuge 24D, Labnet International, USA) for 5 min at 200 g in order to remove titanium (i.e. heavy metal) particles.

Liposome formulations were fed into a Büchi Mini Spray Dryer B-290 equipped with a high performance cyclone (Büchi Laboratories, Switzerland) and a 0.7 mm nozzle. The following conditions were maintained throughout the process, including: an inlet temperature of 100°C (an outlet temperature of 50 ± 3°C), feed rate of 5–6 ml/min, spray flow rate of 400 L/h, with a setup of 100% aspirator. Liposome suspensions were continuously stirred, and simultaneously fed into the spray dryer in order to maintain homogeneity of the suspension, during spray drying. Dry proliposome spray dried powders were transferred from the collecting chamber to an air-tight glass bottle (100 ml) in a desiccator for further studies. Following spray drying, the production yield was calculated using the following Eq. 4 (51).

4 $$\mathrm{Yield}\ \left(\%\right)=\left(\frac{\ \mathrm{W}1}{\mathrm{W}2}\right)\times 100$$

Where, W_1_ is the final weight of formulation collected in the collecting chamber of spray drying and W_2_ is the initial weight of proliposome formulation before hydration in DW (i.e. as a starting material).

### Proliposome Tablet Manufacturing

Proliposome powders were compressed into proliposome tablets using a Stylcam 2000R compaction simulator (Medelpharm, France) fitted with a 7 mm flat punch and die set with 8.98 mm filling height. The selected formulations were typically weighted between 80 and 250 mg. An average compression force of 10 kN with a compression speed of 10 tablets per min was maintained throughout the tabletting process. An ambient temperature of 17°C with relative humidity of 40% was maintained throughout the process.

### Quality Control Tests for Proliposome Tablets

Following tabletting, the selected formulations were subjected to quality control testing. Four different quality control tests were employed to assess these tablet in accordance with BP (British Pharmacopeia) requirements, these were: Weight uniformity, crushing strength, thickness and disintegration testing.

For the weight uniformity test, 20 tablets were randomly selected from each formulation and individually weighed. The mean weight of each batch of proliposome tablets was calculated in accordance with Eq. 5 and the variance of each tablet from the mean was calculated (52).

5 $$\mathrm{Average}\ \mathrm{weight}=\left(\frac{\mathrm{X}1+\mathrm{X}2+\mathrm{X}3+\mathrm{X}5\dots \dots \mathrm{X}20\ }{20\ }\right)$$

For crushing strength, a tablet tester (Dr Schleuniger/ Pharmatron, USA) was employed to measure the mechanical integrity of randomly selected 10 proliposome tablets. Crushing strength of the proliposome tablets were measured in Newton. Thickness of the proliposome tablets was measured using an electronic digital calliper (Copley Scientific, UK). For this study, 20 proliposome tablets were randomly selected and the thickness of the tablets were measured in millimetre (mm). Thickness was assessed in accordance with BP acceptance criteria ±5%; for tablets with a thickness < 12.55 mm or 3% for tablets with a thickness > 12.55 mm (53).

For the disintegration test, 6 tablets were randomly selected from each batch (PTZ Pharma test instruments, Germany). Testing was conducted in a medium of distilled water (900 ml) at a temperature of 37°C (54); with complete disintegration of tablets determined as the disintegration time noted for all six proliposome tablets in min.

### Nebulization Studies Via Two Stage Impinge

A Two Stage Impinge (TSI) was employed as an *in-vitro* lung model for the aerosolization of liposome suspension testing two different nebulizer; an Omron (Vibrating mesh) nebulizer and Ultrasonic nebulizer. The TSI is comprised of an upper stage (Stage 1) representing the upper airway, and the lower stage (Stage 2) representing the lower airway of the respiratory tract. The cut-off aerodynamic diameter between the two stages of TSI is 6.4 μm, hence particle less than this size can deposit in the lower stage of TSI and are referred to as “respirable or fine particle fraction” (55,56). The air flow rate was adjusted to 60 L/min with previously placed 7 ml of DW in the upper stage and 30 ml of DW in the lower stage of TSI. It is the first study where two different nebulizers (i.e. Ultrasonic and Vibrating mesh) were employed for highlighting various features with the help of TSI including; nebulization and sputtering time, mass output, output rate as well as vesicles size determination in various compartment of nebulizer reservoir, upper stage and lower stage of TSI. Moreover, the usage of TSI was implemented to differentiate between nebulizers performance. The TSI is approved by British Pharmacopeia, United State Pharmacopeia and European Pharmacopeia as a standard model for the analysis of nebulized aerosols, thus it was included as a measure of performance (57).

Proliposome tablets were hydrated in nebulizer reservoir (5 ml; prepared with the set ratio of 30 mg/ml) and located in front of TSI mouthpiece. Post nebulization; nebulization time, sputtering time, aerosol mass output (%) aerosol output rate (mg/min) as well as vesicles size in nebulizer reservoir, upper stage and lower stage of TSI were determined for all selected formulations and for both nebulizers (Ultrasonic and Omron) (“Nebulization Performance Determination” section). Aerosol mass output (Eq. 6) and aerosol output rate (Eq. 7) were determined with the aid of following equations;

6 $$\mathrm{Mass}\ \mathrm{output}\ \left(\%\right)=\left(\frac{\mathrm{Weight}\ \mathrm{of}\ \mathrm{nebulized}\ \mathrm{formulation}\ }{\mathrm{Weight}\ \mathrm{of}\ \mathrm{formulation}\ \mathrm{present}\ \mathrm{in}\ \mathrm{the}\ \mathrm{nebulizer}\ \mathrm{prior}\ \mathrm{to}\ \mathrm{nebulization}\kern0.5em }\right)\mathrm{X}100$$

7 $$\mathrm{Aerosol}\ \mathrm{output}\ \mathrm{rate}\ \left(\mathrm{mg}/\min \right)=\left(\frac{\mathrm{Weight}\ \mathrm{of}\ \mathrm{nebulized}\ \mathrm{formulation}\ }{\mathrm{Complete}\ \mathrm{nebulization}\ \mathrm{time}\ }\right)$$

### Cell Viability/Cytotoxicity Test

MRC-5 cells (Medical Research Council cell strain 5) and immortalized human lung fibroblast SV2 cells were plated into flat-bottom, black 96-well plates at a density of 1 × 104 cells/ml (90 μl per well) using Dulbecco’s Modified Eagle Medium (DMEM) as a growth medium with 10% of Foetal Bovine Serum (FBS), 1% of L-glutamine and 1% of antibiotic-antimycotic solution. These cells (i.e. MRC-5 and MRC5 SV2) were incubated at 37°C in a humidified atmosphere of 5% of CO_2_ for 24 h. After 24 h, these plates containing normal and cancer cells were tested with 10% of F3 formulation (i.e. PTX-free and PTX-loaded formulations for comparison; when 30 mg of proliposome was hydrated in 3 ml of DW), followed by a further incubation of 24 h. On day three, 10% of Alamar Blue (AB) was added to each well (kept at 37°C) to quantify cell viability (after 3 h of incubation with AB, plates were allowed to cool at room temperature). A microplate reader (Epoch, BioTek Instruments Ltd., Swindon, UK) was used for reading plates (fluorescence excitation at 545 nm used, emission at 600 nm) (32). Values obtained for formulation F3-treated well were compared with control wells that were treated with DW. Control values were set at 100% and the values of formulation F3-treated wells were normalized to the control values.

### Statistical Analysis

All experiments were carried out in triplicate and the values were expressed as mean ± standard deviation (SD). For statistical analysis, One-way Analysis of Variance (ANOVA) and student’s *t test* was performed using SPSS software where appropriate to compare more than two groups or two set of data respectively. Statistically, significant differences between the two groups were indicated as a *p value* less than 0.05.

## Results and Discussion

### Morphology of Carbohydrate Carriers for Proliposome Formulations

**Fig. 1:**
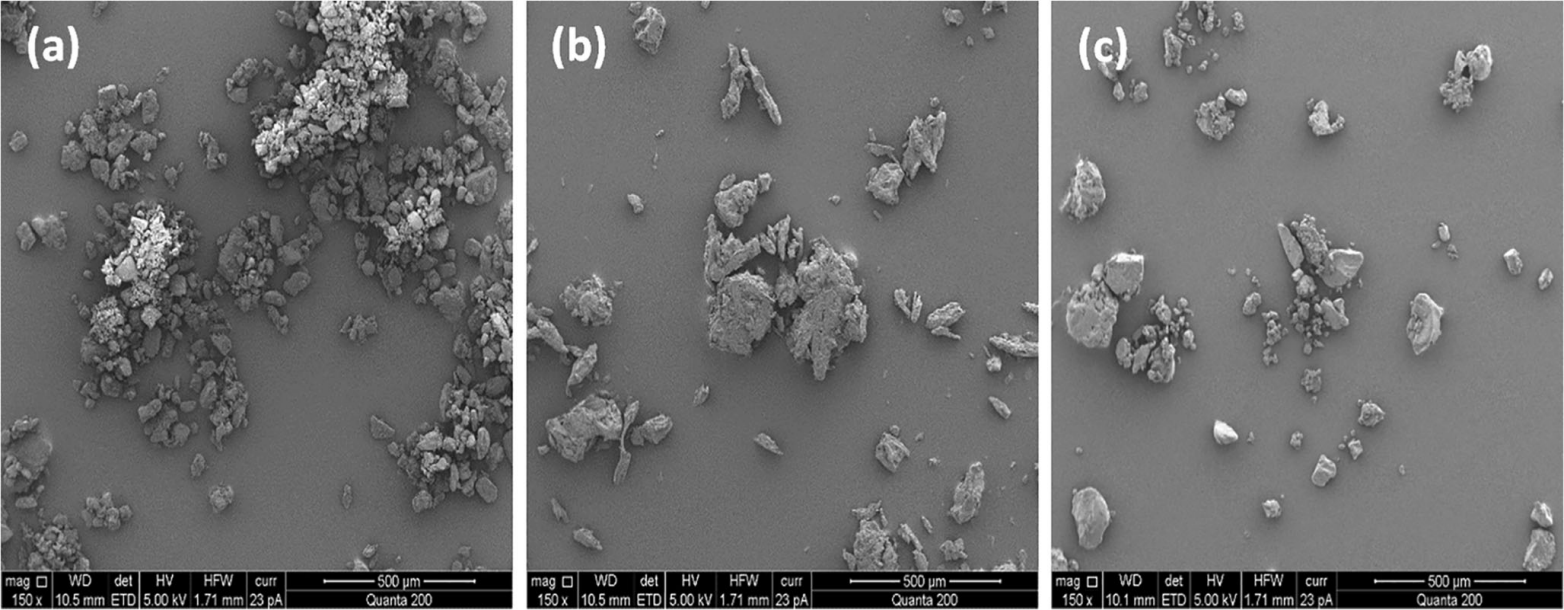
SEM images of coarse carbohydrate including; (**a**) lactose monohydrate (LMH), (**b**) microcrystalline cellulose (MCC) and, (**c**) Starch were employed for proliposome formulations. These images are typical of three such different experiments.

The surface morphology of various carbohydrate carriers i.e. LMH, MCC and Starch were examined using scanning electron microscopy (SEM). LMH was observed to mainly consist of small particles, uniform in size and shape, being typically employed as a filler in the manufacture of tablets via direct compression (58) (Fig. 1a). Upon formulation as proliposomes powder, these particles were non-porous and with uniformly distributed lipid phase a select advantages when compared to other carriers processed using the slurry method (36,59) (please check surface morphology of coarse carbohydrate carrier with high magnification in supplementary data (Fig. b)).

MCC as a carbohydrate carrier is comprised of both crystalline and amorphous regions with a round to oblong in shape (i.e. oblong). Moreover, MCC is known to form aggregates of smaller cellulose fibres (Fig. 1b) (58). This aggregation results in a high degree of variation in particle size (Fig. 1b). MCC particles, based on their shape and size may impact the flowability of particles. However, MCC offers good tablet forming and disintegration properties (58).

Upon examination, Starch particles were irregular in shape and different in size (Fig. 1c). Starch offer sufficient disintegration properties by swelling dramatically during water uptake (58).

### Flowability Studies of Proliposome Powder

Upon analysis, amongst the coarse carbohydrate carriers, LMH exhibited *good* flowability in accordance with Carr’s scale of flowability (14.66 ± 3.05) (*p < 0.05*) when compared to the *passable* and *fair* flowability of MCC and Starch (22.53 ± 0.28 and 19.30 ± 2.60) respectively (Tables II and III). These coarse carrier values were used as a control in order to assess the impact of the incorporation of various phospholipids (i.e. SPC, HSPC and DMPC) or lipid phase to carrier ratios (1:5, 1:15 and 1:25 *w*/w) to manufacture PTX-loaded proliposome. Irrespective of phospholipid type and lipid phase to carrier ratio, no significant differences (*p > 0.05*) in flowability was observed in the presence or absence of PTX and therefore formulations without PTX were not included.

**Table III:** Coarse Carbohydrate Carriers (i.e. LMH, MCC and Starch) Employed to Prepare Proliposome Powder Formulations (F1 – F27) Followed by Their Characterizations via Compressibility Index, Angle of Repose, Liposome Size, Pdi, Zeta Potential and Entrapment Efficiency. Data are Mean ± SD, *n* = 3

Formulations name	Compressibility index (%)	Angle of repose (°)	Size (μm)	PDI	Zeta potential (mV)	Entrapment efficiency (%)
LMH	14.66 ± 3.05	10.57 ± 1.23	N/A	N/A	−4.09 ± 0.52	N/A
MCC	22.53 ± 0.28	13.16 ± 1.11	N/A	N/A	0.24 ± 0.80	N/A
Starch	19.29 ± 2.60	13.46 ± 1.24	N/A	N/A	−13.20 ± 3.42	N/A
F1	9.19 ± 0.63	20.45 ± 0.80	6.25 ± 0.71	4.54 ± 1.23	−3.55 ± 0.95	96.68 ± 3.68
F2	13.87 ± 0.14	19.62 ± 0.99	6.66 ± 0.68	3.57 ± 0.75	−3.61 ± 0.85	95.45 ± 3.16
F3	14.81 ± 0.36	17.24 ± 0.43	5.35 ± 0.76	3.32 ± 0.88	−3.10 ± 1.51	95.45 ± 2.78
F4	9.50 ± 0.31	20.47 ± 1.01	5.78 ± 0.65	3.33 ± 0.79	−4.52 ± 1.23	95.99 ± 2.92
F5	11.66 ± 1.12	18.62 ± 0.54	6.46 ± 0.69	4.32 ± 1.26	−4.83 ± 1.25	96.55 ± 4.68
F6	15.01 ± 0.35	16.41 ± 0.52	6.27 ± 0.59	2.94 ± 0.82	−3.74 ± 0.52	96.69 ± 4.78
F7	9.13 ± 0.43	23.92 ± 1.45	5.89 ± 0.66	3.53 ± 0.79	−2.74 ± 0.88	94.99 ± 3.77
F8	12.40 ± 0.61	19.96 ± 1.35	6.04 ± 0.74	2.72 ± 0.97	−3.38 ± 0.98	93.88 ± 5.69
F9	14.56 ± 0.14	15.16 ± 0.72	5.43 ± 0.68	2.54 ± 0.89	−2.49 ± 1.91	93.56 ± 4.78
F10	19.85 ± 0.65	23.54 ± 1.41	9.52 ± 0.89	3.35 ± 0.55	−3.42 ± 1.20	96.41 ± 3.88
F11	21.84 ± 0.99	21.33 ± 2.39	10.69 ± 1.26	3.38 ± 0.69	−3.09 ± 1.29	95.99 ± 3.92
F12	22.65 ± 0.42	19.26 ± 0.46	10.17 ± 1.42	2.79 ± 0.76	−3.18 ± 1.41	97.05 ± 4.75
F13	19.94 ± 0.94	24.32 ± 2.56	9.35 ± 0.93	2.83 ± 0.68	−4.67 ± 1.46	96.68 ± 5.55
F14	20.98 ± 1.04	21.42 ± 0.66	9.34 ± 1.05	4.22 ± 1.06	−4.65 ± 1.21	95.91 ± 5.70
F15	20.51 ± 2.44	19.21 ± 4.26	9.05 ± 1.26	3.88 ± 0.67	−4.77 ± 1.32	96.84 ± 5.48
F16	18.06 ± 2.35	21.66 ± 1.73	8.96 ± 0.85	2.64 ± 0.77	−3.99 ± 1.35	93.87 ± 4.56
F17	20.74 ± 2.76	21.74 ± 1.28	9.39 ± 1. 02	4.45 ± 1.16	−3.48 ± 0.91	94.08 ± 4.55
F18	24.06 ± 0.47	18.01 ± 0.64	8.53 ± 1.36	2.47 ± 0.77	−2.58 ± 1.39	93.27 ± 5.44
F19	18.36 ± 1.43	25.41 ± 1.81	9.59 ± 1.22	4.92 ± 1.76	−3.33 ± 1.04	97.01 ± 6.25
F20	22.32 ± 1.24	25.29 ± 2.79	10.01 ± 1.45	2.41 ± 0.98	−3.01 ± 0.71	96.91 ± 6.19
F21	23.67 ± 1.41	20.69 ± 2.15	12.07 ± 2.09	2.65 ± 0.65	−3.09 ± 0.85	96.73 ± 4.09
F22	19.24 ± 1.38	24.71 ± 1.56	9.04 ± 1.06	4.43 ± 1.16	−4.22 ± 1.14	96.46 ± 5.90
F23	23.83 ± 1.05	22.87 ± 1.72	10.74 ± 1.52	2.86 ± 0.72	−4.11 ± 1.12	95.65 ± 5.73
F24	24.95 ± 1.55	17.93 ± 0.53	7.84 ± 0.97	2.45 ± 0.91	−3.88 ± 0.91	97.12 ± 2.96
F25	18.77 ± 1.08	25.11 ± 0.52	10.85 ± 1.53	3.88 ± 0.76	−2.93 ± 0.96	94.19 ± 6.75
F26	22.66 ± 1.33	24.88 ± 2.36	12.28 ± 2.45	4.83 ± 1.46	−3.83 ± 0.97	93.88 ± 4.68
F27	23.89 ± 0.30	20.67 ± 2.64	11.15 ± 2.05	3.95 ± 1.01	−2.96 ± 0.95	94.77 ± 4.25

Upon using LMH with all three phospholipid formulations (F1 – F9), F3, F6 and F9 exhibited a *good* flowability (*p < 0.05*) (14.81 ± 0.36 and 15.01 ± 0.35 and 14.56 ± 0.14); compared *excellent* flowability of F1, F4 and F7 (9.19 ± 0.63, 9.50 ± 0.31 and 9.13 ± 0.43) respectively (Table III). Proliposome powders prepared from LMH as a carbohydrate carrier (F1 – F9) for powder flowability studies, compressibility index exhibited enhanced powder flowability for 1:5 > 1:15 > 1:25 *w*/w (irrespective of phospholipid type) (Table III). Similar trends were noted for MCC and Starch-based proliposome powder formulations, where compressibility index was low for 1:5 and higher for 1:25 formulations (F12, F15 and F18); considered as *fair* flowability for 1:5 and *passable* for 1:25 formulations.

Superior flowability is advantageous in die filling, enhancing the uniformity in terms of tablet weight for PTX-loaded proliposomes during manufacture. Higher lipid content, due to the presence of higher phospholipid amount (i.e. 1:5 *w*/w) in proliposome formulations associated with more favourable compressibility index values (i.e. very low volume change noted in the graduated cylinder prior and post tapping). This was associated with a thicker coating of lipid phase (due to the presence of phospholipid) on the carrier surface; this elevated lipid concentration my reduce particle flow and subsequently restrict tapped density. Lower lipid concentrations in proliposome formulations (e.g. 1:25 *w*/w formulations) were associated with reduced resistance, thus particle flowability was notably different. These results are in agreement with previous research (17), where Stewart assay quantitative analysis of phospholipid in 1:5, 1:10 and 1:15 *w*/w formulations (i.e. phospholipid to carrier ratios w/w) demonstrated that phospholipid concentration in 1:5 *w*/w formulations were more when compared to the phospholipid concentration in 1:10 and 1:15 w/w formulations (17). Therefore, it was suggested that higher lipid content in formulation is associated with thicker coat, whereas lower lipid concentration with thinner/inconsistent coat over carbohydrate carriers change (please check supplementary data (Fig. c)) (59). Thus, lower and higher changes in volume for 1:5 and 1:25 w/w powder formulations were observed in a graduated cylinder. Additionally, thicker coat of phospholipid on carbohydrate carrier may enable proliposome powder sticking/picking to the punches and die cavity during tabletting, responsible for variation in tablets weight as well as tablets morphology.

Overall, it was found that proliposome powder with 1:5 w/w ratios exhibited better flowability but due to the higher resistance caused by phospholipid content may explain that 1:5 w/w formulation ratio are actually don’t show the real flowability of powder. This point was further explained by AOR studies. On comparison of all the carriers at the 1:25 w/w lipid to carrier ratio, LMH exhibited good flowability, whereas the tested counterparts (MCC and starch) exhibited passable flowability. The reduced lipid concentration at this ratio, may be responsible for improving flow properties when compared to formulations with a higher lipid concentration.

Coarse carbohydrate carriers exhibited *excellent* flowability via AOR for LMH, MCC and Starch powders (Table III) (47). When using LMH as a carrier for proliposome powder formulations, *excellent* flowability was noted for all prepared formulations (F1 – F9), with the lowest (*p < 0.05*) AOR was elicited by F3, F6 and F9 (17.24 ± 0.43°, 16.41 ± 0.52° and 15.16 ± 0.72°) formulations respectively, when compared to the alternative LMH proliposome formulations (Table III). LMH has also been identified to possess higher flowability and compressibility when direct compression is used for tablet manufacturing (58). The *excellent* flowability of LMH as a carbohydrate powder may be attributed to the uniform size distribution (“Morphology of Carbohydrate Carriers for Proliposome Formulations” section), identifying it as a suitable carrier for proliposome formulations (17).

MCC and Starch-based proliposome formulations also exhibited an *excellent* AOR with respect to Carr’s scale (Table II) (47). Upon investigation of AOR amongst F10 – F27, MCC-loaded formulation F18 (18.01 ± 0.64°) and Starch-loaded formulation F24 (17.93 ± 0.53°) exhibited lower angle values when compared to the remaining formulations (F10 – F27). MCC as a carbohydrate carrier is observed to be oblong shape particles with asymmetrical shape and size distribution, which can be observed in the surface morphology via SEM (“Morphology of Carbohydrate Carriers for Proliposome Formulations” section). It is proposed, that as a result of this shape, MCC particles demonstrated greater resistance to powder flow, thus formulations based on these carrier exhibited higher AOR values (60). Starch particles were noted to be irregular and varied in size and shape (Fig. 1).

Overall, it was determined that compressibility index was lower for 1:5 *w*/w formulations ratios when compared to 1:15 and 1:25 w/w (regardless of phospholipid type). This was attributed to the high concentration of lipid phase (i.e. 1:5 w/w), suggested greater resistance to powder flow during tapping and therefore deceptive enhanced flowability was achieved. However, AOR finding suggested that formulation with 1:25 w/w lipid phase to carrier ratio are better in terms of flowability (i.e. low angle) and would demonstrate uniform die filling. Therefore, for both compressibility index and AOR, formulations with low lipid phase (1:25 w/w) may be of greater suitability in terms of flowability to manufacture tablets.

### Characterization and Analysis of Liposome Size, PDI, Zeta Potential and PTX Entrapment

Liposome size was measured following vesicle generation via hydration and annealing from proliposome powders. Size of liposomes prepared from LMH, MCC and Starch-based proliposome powder ranged between 5.35 ± 0.76–12.28 ± 2.45 μm (Table III). LMH-based proliposome formulations (F1 – F9) liposome generation were consistent (*p > 0.05*) in size, ranging from 5.35 ± 0.76–6.66 ± 0.68 μm; this may be attributed to the uniform carrier size and shape, as lipid phase equally distributed over the LMH carrier particles (“Morphology of Carbohydrate Carriers for Proliposome Formulations” section) (Table III). Comparatively, a significant difference (*p < 0.05*) in size was noted between LMH-based liposomes and both MCC and Starch-based liposomes (Table III). These results are suggestive that liposomes prepared from LMH-based proliposomes are superior in terms of consistency in size, when compared to liposomes generated MCC and Starch-based proliposomes. Moreover, a hypertonic environment outside liposome vesicles was created by the slow dissolution of LMH (i.e. enable water movement from inside vesicles towards outside); that may possibly shrink liposome as compared to liposomes generated from MCC or Starch-based proliposomes (59).

Upon investigation, no significant difference (*p > 0.05*) was observed with respect to PDI values between liposomes hydrated from LMH, MCC and Starch-based proliposome formulations using Malvern Mastersizer. The minimum and maximum values ranged between 2.41 ± 0.64–4.83 ± 1.46 (Table III). Values from size distribution exhibited a hetero dispersed size, irrespective carrier or phospholipid type used (Table III). This trend was mirrored in terms of Zeta potential, with no significant difference (*p > 0.05*) observed for liposome formulations (F1 – F27) via Malvern Zetasizer. Zeta potential values ranged from −2.49 ± 1.91 to −4.83 ± 1.25 for all formulations (Table III).

Liposomes were prepared via hydration from LMH, MCC and Starch-based proliposomes formulations (F1 – F27). Entrapment efficiency was found to range from 93.87 ± 4.56–97.12 ± 2.96% respectively, with no significant difference noted (*p > 0.05*) amongst all formulations (F1 – F27) (Table III). PTX is hydrophobic drug and therefore embedded in bilayers of liposomes but due to its bulkiness and asymmetric nature, its loading in liposome is challenging. Various studies demonstrated that PTX maximum loading in liposomes is ~ ≤ 3.3 mol% of the phospholipid component (61,62). Whereas, higher loading of PTX in liposomes higher than this percentage may lead to leakage after preparation or during storage (23). Therefore, 2 mol% of PTX was employed in these formulation and therefore have shown high drug entrapment in liposomes. Similarly, high entrapment of PTX in liposome vesicles was also reported by You *et al*. with the entrapment ranging from 90.29% – 91.65% (63). Additionally, 94% of PTX entrapment was also demonstrated by Rane and Prabhakar (64), and 70.83 ± 0.78% – 89.26 ± 3.05% using SPC 100 (65). Higher entrapment efficiency in all formulations may be attributed to the presence of long hydrocarbon chains of the phospholipid component i.e. SPC, HSPC and DMPC, which may be able to entrap or accommodate higher PTX proportions in the bilayers of liposomes as compared to the phospholipid with shorter hydrocarbon chain length. Phospholipid with shorter chain length offer low drug accumulation and drug loading capacity and therefore higher amount of drug may present in formulation as unentrapped part (i.e. free drug). It was also reported that longer hydrophobic inner core can be created by the long acyl chain to entrap PTX (66) as well as using DMPC as a phospholipid (23,28,67).

Overall, liposomes generated from LMH-based proliposome formulations offered the highest degree of consistency in size (F1 – F9) when compared to proliposome formulations based on other carriers (i.e. MCC and starch) (F10 – F27). PDI values were consistent for all liposome formulations irrespective of phospholipid or carbohydrate carrier type. Zeta potential values were low (*p > 0.05*) when liposome were generated from DMPC, whereas a marginal increase was observed in the negative scale for SPC and HSPC formulations (due to the purity of phospholipid); this increase (*p > 0.05*) was independent of carbohydrate carrier type. Entrapment efficiency achieved was in excess of 93% for all liposome generated from proliposome formulations (please check PTX chromatogram in supplementary data (Fig. d)).

### Formulation Selection

Post-examination of all 27 formulations (employing compressibility index, AOR, size, PDI, Zeta potential and PTX entrapment efficiency studies); four formulations were selected based on superior properties based on the aforementioned parameters and put forward for further investigation. With respect to compressibility index and AOR studies, LMH formulated in low lipid phase and high carrier ratio elicited desirable results (i.e. F3, F6 and F9) (“Flowability Studies of Proliposome Powder” section). Size analysis indicated that the size of liposome vesicles was comparatively smaller (*p > 0.05*) when LMH was employed as a carbohydrate carrier regardless of phospholipid selection (“Characterization and Analysis of Liposome Size, PDI, Zeta Potential and PTX Entrapment” section). Moreover, formulation F24 also exhibited lower vesicles size, which are closer to the size generated for LMH formulations. PDI and Zeta potential analysis demonstrated no significant difference (*p > 0.05*) for all formulations (i.e. F1 – F27) ((“Characterization and Analysis of Liposome Size, PDI, Zeta Potential and PTX Entrapment” section). Similar results of no significant difference (*p > 0.05*) between formulations were found for PTX entrapment efficiency, where the entrapment was in excess of 93% for all formulations (F1 – F27) regardless of phospholipid type (SPC, HSPC and DMPC) or carbohydrate carrier type (LMH, MCC and Starch).

On the basis of above analysis and investigation, formulations F3, F6, F9 and F24 were selected for further studies.

### TEM of Liposomes

**Fig. 2:**
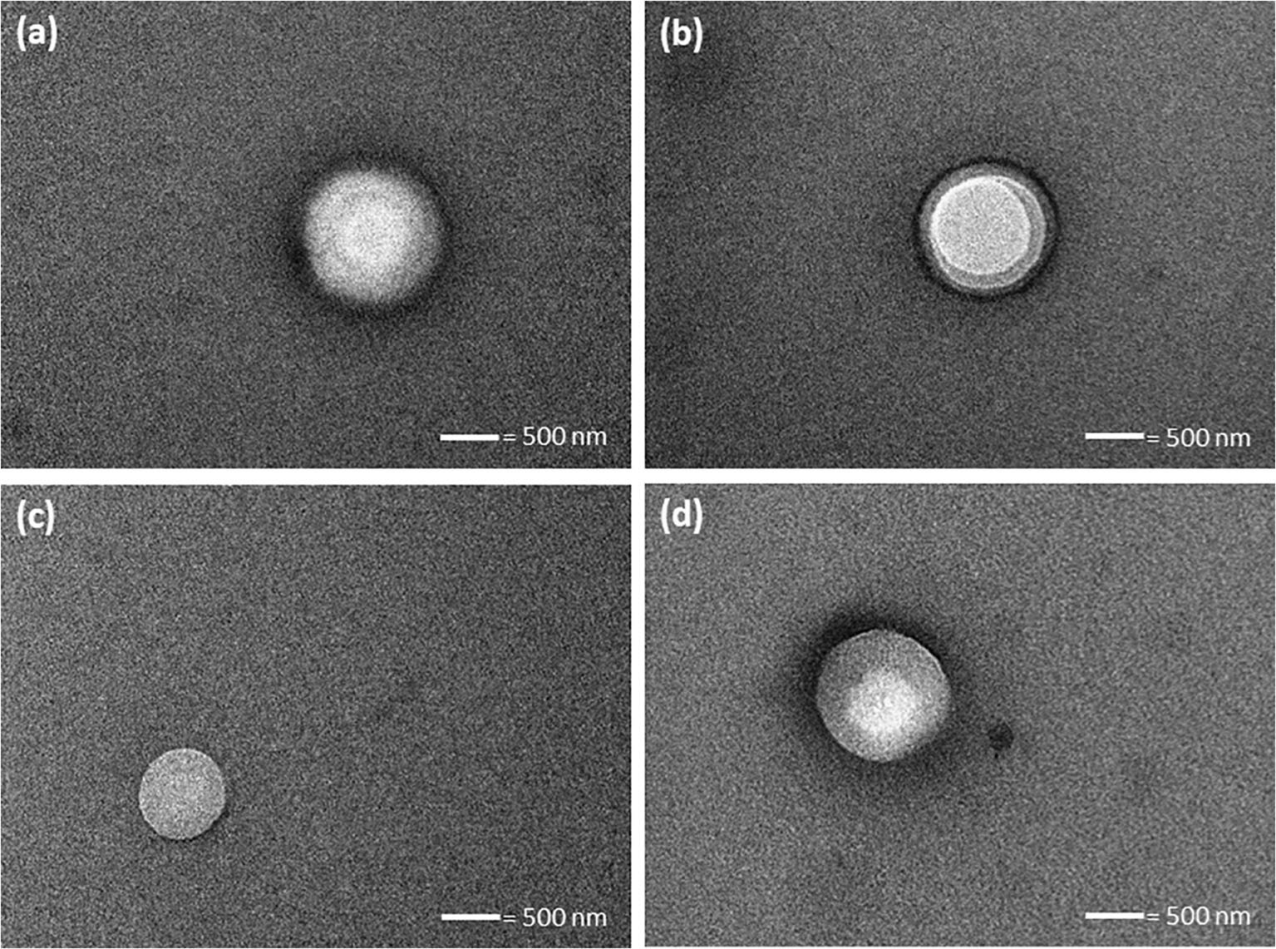
TEM images of liposomes generated from (1:25 *w*/w lipid phase to carrier ratio) LMH-based proliposome containing (**a**) F3, (**b**) F6, (**c**) F9 and, Starch-based proliposome (**d**) F24. These images are typical of three such different experiments.

TEM was employed in order to determine the morphology and architecture of formed liposomes following hydration of LMH and Starch-based proliposome powders. Liposomes were observed to be spherical in shape. These vesicles may be multilamellar or possibly unilamellar liposomes (Fig. 2). These images confirm that liposomes were successfully generated from the proliposome formulations.

### Characterization of Nano Formulations Via Probe Sonication Prior and Post Spray Drying

Amongst the 27 formulations, F3, F6, F9 and F24 were selected and referred as Micro (M) formulations due to the size of vesicles in micrometre range (i.e. used for further studies). These formulations were converted into Nano (N) vesicles via probe sonicated followed by spray drying to obtain the dry product and therefore these spray dried (SD) formulations were denoted as spray dried Nano (SDN) formulations.

LMH liposomes were significantly reduced (*p < 0.05*) to 355.41 ± 7.54, 364. 89 ± 8.75 and 349.66 ± 7.91 nm for formulations F3, F6 and F9, when compared to Starch-based liposomes 750. 84 ± 10.48 nm (i.e. F24) (Table IV). This may be attributed to the carbohydrate carrier Starch, which possesses swelling properties and may offer resistance to the size reduction process (58).

**Table IV:** Size, Polydispersity Index and Entrapment Efficiency (PTX) of Liposome Vesicles were Compared after Probe Sonication (PS) and Generation of Liposome from Spray Dried Nano (SDN) Formulations (F3SDN, F6SDN, F9SDN and F24SDN). Spray Dried Formulations were Also Analysed for Production Yield and Compressibility Index. Data are Mean ± SD, *n* = 3

Formulations	Size (nm)		Polydispersity index		Production yield (%)	Compressibility index (%)	Entrapment efficiency (%)	
	After PS	After SD	After PS	After SD	After SD	After SD	After PS	After SD
F3SDN	355.41 ± 7.54	368.94 ± 9.75	0.324 ± 0.57	0.345 ± 0.61	56.05 ± 2.75	35.42 ± 3.61	81.45 ± 5.76	73.66 ± 7.21
F6SDN	364. 89 ± 8.75	370.61 ± 13.87	0.297 ± 0.39	0.337 ± 0.53	56.14 ± 1.69	26.96 ± 6.91	75.26 ± 5.66	69.55 ± 6.48
F9SDN	349.66 ± 7.91	356.56 ± 14.85	0.342 ± 0.48	0.364 ± 0.52	53.05 ± 3.48	31.67 ± 4.85	77.13 ± 6.77	69.21 ± 5.92
F24SDN	750. 84 ± 10.48	973.82 ± 16.85	0.372 ± 0.64	0.384 ± 0.62	38.74 ± 1.06	46.55 ± 6.45	74.91 ± 5.71	65.88 ± 6.85

The aforementioned four Nano sized liposome formulations were SD following hydration, forming a dry proliposome powder. These formulations were then referred to as F3SDN, F6SDN, F9SDN and F24SDN respectively. Following rehydration of these SDN proliposome powder formulations, size was observed to increase (*p > 0.05*) to 368.94 ± 9.75, 370.61 ± 13.87, 356.56 ± 14.85 and 973.82 ± 16.85 nm for F3SDN, F6SDN, F9SDN and F24SDN (Table IV). Similarly, no significant differences (*p > 0.05*) were observed in terms of PDI (polydispersity index) values of liposomes post-probe sonication in addition to following rehydration of SD formulations.

Upon analysis, PTX entrapment was significantly decreased (*p < 0.05*) using probe sonication when compared to entrapment before sonication (Tables III and IV). This decrease in entrapment efficiency was associated with high frequency vibration/energy which reduced liposomes particle size and hence enabled drug leakage. Similar results were also reported in the literature, which demonstrated a decrease in entrapment efficiency after probe sonication (68,69) as well as extrusion (38). The reconstitution of Nano particles from powder formulation (spray dried formulation) has been demonstrated to further decrease entrapment (*p > 0.05*) (70), which may be related to the formulation lost during spray drying process. However, no significant difference (*p > 0.05*) was demonstrated in the entrapment efficiency of PTX after probe sonication and the rehydration of spray dried formulation (Table IV).

The production yield of SDN proliposome powders collected post spray drying ranged from 38.74 ± 1.06% to 56.14 ± 2.65% for all four formulations (Table IV). The lowest yield (*p < 0.05*) observed was for the F24SDN formulation (i.e. 38.74 ± 1.06%) (Starch-based proliposome) when compared to LMH-based proliposome (F3SDN, F6SDN and F9SDN) formulations (Table IV). The reduced yield of Starch-based formulation may be related again to its swelling property during hydration. Upon spraying the high density Starch droplets/particles may deposit and adhere to the internal walls of drying chamber; hence a lower concentration of formulation may dry in the air and collect in the collecting chamber of spray drying apparatus. Moreover, it is also possible that the inlet temperature employed, may cause insufficient droplet/particle drying within the drying chamber. High moisture content of the resultant particles may be a causative factor of greater adherence to the walls of drying chamber as well as cyclone chamber, lowering production yield (71,72).

Following spray drying, compressibility index values were generated for all four formulations (i.e. F3SDN, F6SDN, F9SDN and F24SDN). High compressibility index values ranged from *poor* for F6SDN (26.96 ± 6.91) to *very poor* for both F3SDN and F9SDN (35.42 ± 3.61 and 31.67 ± 4.85) to a *very very poor* values for F24SDN (46.55 ± 6.45) (Tables II and IV). High compressibility index values were associated with the fluffy nature and surface phenomenon i.e. cohesiveness of SD powders, causing sticker during powder flowability. Interestingly, upon tapping, spray dried powders compressed more readily than standard proliposome powders. This may attribute to the small particle size, and improved morphology (i.e. particles spherical shape) when compared to coarse carbohydrates or proliposome powders (60).

Consequently, formulation F24SDN was eliminated from further studies of proliposome tableting and nebulization, due to its high compressibility index values (i.e. 46.55 ± 6.45; *very, very poor*) and low production yield (i.e. 38.74 ± 1.06) (Table IV). A counterpart of this formulation, F24 was also eliminated due to high compressibility index value (i.e. 24.95 ± 1.55; *passable*) and large size of vesicles (i.e. 7.84 ± 0.97 μm) (Table II). Based on percentage yield and size; F3SDN, F6SDN and F9SDN were selected. However, these formulations also exhibited compressibility index values, which may possibly not enough to compress well due to the high coherence and poor flowability properties (60). In summary, the following formulations were further investigated for tablet manufacture: F3, F6, F9, F3SDN, F6SDN and F9SDN.

### Manufacture and Quality Control Tests of PTX-Loaded Proliposome Tablets

Following selection of the proliposome Micro formulations (F3, F6, F9) and SD Nano formulations (F3SDN, F6SDN and F9SDN), these were subjected to tableting via a Stylcam 2000R compaction simulator (Medelpharm, France) (please check proliposome tablets in supplementary data (Fig. e)). These selected formulations have used LMH as a carbohydrate carrier in a 1:25 *w*/w lipid phase to carrier ratio.

#### Weight Variation Test

In accordance with BP requirements for coarse tablets, the criteria set for uniformity of weight is that; if the weight of tablet is greater than 80 mg and less than 250 mg, to comply with the test; not more than two individual tablet weight should deviate from the mean weight by more than ±7.5%. Moreover, none of the tablets should deviate by more than twice that percentage i.e. ±15% (52). Proliposome Micro formulations i.e. F3, F6 and F9 had an average weight ranging between 129.40 ± 3.85–159.58 ± 4.99 mg (Table V). Similarly, the weight variation for SD Nano formulations (F3SDN, F6SDN and F9SDN) were found to be in the range of 90.15 ± 5.24 mg and 126.55 ± 5.06 mg (Table V). According to the acceptance range of ±7.5%; all tablets were within the range for F3, F6 and F9 formulations, passing a set standard of BP (52).

**Table V:** PTX-Loaded Micro Proliposome (F3, F6 and F9) and SDN Formulations (F3SDN, F6SDN and F9SDN) Tablet Characterization via Uniformity of Weight (mg), Crushing Strength (N), Thickness (mm) and Disintegration Time (min) in Accordance with BP. Data are Mean ± SD, *n* = 3

Formulations	Weight uniformity (mg)	Crushing strength (N)	Thickness (mm)	Disintegration time (min)
F3	129.40 ± 3.85	14.08 ± 1.95	2.33 ± 0.51	14.35 ± 0.56
F6	159.58 ± 4.99	25.60 ± 2.61	2.93 ± 0.56	20.05 ± 1.02
F9	142.80 ± 3.57	22.85 ± 2.65	2.43 ± 0.11	17.45 ± 1.24
F3SDN	90.15 ± 5.24	7.60 ± 1.57	1.24 ± 0.23	10.57 ± 2.14
F6SDN	126.55 ± 5.06	9.80 ± 1.24	1.96 ± 0.13	14.89 ± 2.57
F9SDN	109.16 ± 4.13	8.06 ± 1.15	1.80 ± 0.23	12.51 ± 1.65

Comparatively, in SD tablet formulations F3SDN, F6SDN and F9SDN, five tablets demonstrated a deviation of ±7.5% in weight for F3SDN. Whereas four tablets deviated from the set standard for F6SDN and F9SDN formulations. The BP specifies that only two tablets are allowed to pass from the range of ±7.5% (Table V). Tablets weight variation for F3SDN, F6SDN and F9SDN may be attributed to the low flowability and high fluffy nature of SD powder (60). It is noteworthy that post spray drying, the resultant powder becomes electrostatically charged resulting in particle cohesion/adhesion, thus developing a fluffy nature, compromising smooth powder flow properties. The low level of powder flowability i.e. *poor* to *very poor* was determined for post spray drying (Table IV), which indicated SD powder to demonstrate resistance to flow and with the powder thus unable to fill the die cavity with consistent weight of powder (i.e. already adjusted), leading to inconsistent weight of tablets. Similar results of electrostatic charge particles post spray drying were also reported in the literature (73,74). Similar results were also found for the compressibility index values for F6SDN, which indicated *very poor* flowability of powder, whereas F3SDN and F9SDN exhibited *very very poor* powder flow (Tables II and IV) (47).

Thus, proliposome Micro proliposome formulations F3, F6 and F9 passed the uniformity of weight test, whereas proliposome SDN formulations F3SDN, F6SDN and F9SDN failed the test.

#### Crushing Strength

Proliposome Micro formulations F3, F6 and F9 exhibited high crushing strength (Table V). SD Nano formulations tablets (F3SDN, F6SDN and F9SDN) showed comparatively lower crushing strength (*p < 0.05*) when compared to Micro formulations tablets (F3, F6 and F9) (Table V). This difference may be associated with physical differences between SD and coarse LMH. Coarse LMH exhibits superior compactability as well as good flowability due to its small uniform particle size and shape (58). Moreover, the high cohesiveness of SD formulation particles may result in poor flowability, occupying irregular spaces in the die cavity of tabletting machine, requiring a lower force of compression when similar compression conditions were used for all formulations.

Thus the lower crushing strength exhibited by tablets formed from proliposome SD Nano formulations are more susceptible to breakage during handling and shipping, whereas proliposome Micro formulation tablets are stronger and would retain integrity of a solid dosage form.

#### Thickness and Disintegration Time of Tablets

Similar to crushing strength, thickness of Micro formulation tablets were higher (*p < 0.05*), when compared to SD N formulation tablets (Table V). This difference is attributed to the nature of SD proliposome powders (as explained earlier in “Weight Variation Test” section and “Crushing Strength” section), which exhibited *very poor* powder flowability leading to variation in tablet weight, reflected in tablet thickness. The reduced flowability of SD powder formulations may therefore be responsible for irregular filling of tabletting die cavity resulting in differences in tablet thickness.

Disintegration time also varied significantly (*p < 0.05*) between formulations F3, F6 and F9 (Table V). Elevated disintegration times for F6 and F9 were directly related to the uniformity of tablet weight and also reflected via crushing test. Compression force is typically adjusted and set to apply a specified force and distance in the die to compress powder into tablet form. With increased mass of powder in the tabletting die, a higher compression force is utilized to compress the powder into tablets (“Weight Variation Test” section). In terms of uniformity of weight, F6 exhibited a higher tablet weight followed by F9 and subsequently lower weight was elicited by F3. Overall, a higher compression force was employed to compress F6 proliposome powder formulation when compared to F9 and F3 respectively. Similar findings were noted in terms of crushing strength, where an elevated crushing force was required for F6, followed by a reduced force F9 and a further reduced force for F3 tablets (“Crushing Strength” section). Disintegration time for the formulations mirrored the previous test trends. Disintegration time was longest for F6 the proliposome tablets (i.e. 20.05 ± 1.02 min), followed by F9 (17.45 ± 1.24), with the lowest disintegration time observed for the F3 formulation tablets (i.e. 14.35 ± 0.56). Out of three Micro proliposome tablets formulations (i.e. F3, F6 and F9), only F3 tablets disintegrated within 15 min, whereas F6 and F9 failed the test by longer disintegration time (i.e. 4 tablets from six tablets showed disintegration time more than 15 min) (Table V). According to BP. (54) requirements, the set standard required to pass disintegration test is for all 6 tablets to disintegrate within 15 min. In the event that one or two tablets do not disintegrate within 15 min, the test has to be repeated for 12 more tablets. From this second test, out of all 18 tablets, 16 tablets should disintegrate within 15 min in order for the test to be passed.

For proliposome SD Nano (F3SDN, F6SDN and F9SDN) tablet formulations, the disintegration time was comparatively shorter (*p < 0.05*) (10.57 ± 2.14, 14.89 ± 2.57 and 12.51 ± 1.65 min) than F3, F6 and F9 (Table V). This may be associated with the uniformity of weight and lower crushing strength test results. It is suggested that the lower the tablet weight (via low filling volume) combined with lower compression force (for tablet manufacture) and lower crushing force (employed to break the tablets) ultimately reduced the disintegration time. Moreover, SD powder are typically porous and highly hygroscopic in nature and therefore they tend to absorb water (75,76). When tablets are manufactured from SD powder, they enhance water absorption and thus disintegrate rapidly. All SD proliposome powder tablets disintegrated within the set BP standard (i.e. 15 min).

Overall, SD Nano tablet formulations F3SDN, F6SDN and F9SDN passed the disintegration test, however these tablets were fragile as indicated by crushing strength testing. Moreover, these formulations failed the weight uniformity test. Contrastingly, Micro formulation F3, F6 and F9 passed the weight uniformity test and demonstrated high crushing strength, however only F3 tablet formulation passed the disintegration test. From the BP tests, it was concluded that the F3 Micro proliposome tablet formulation passed the quality control tests and thus was studied for nebulization studies.

### Nebulization Performance Determination

The performance of nebulization was determined using measurements of nebulization time, sputtering time, mass output, output rate, size and PDI of liposome suspensions via Vibrating mesh (i.e. Omron) and Ultrasonic nebulizers.

#### Nebulization of PTX-Loaded Proliposome Tablets

This is the first study where only a minimal number of constituents were used (i.e. phospholipid, cholesterol, drug and carrier) to compress them into tablets when compared to conventional tablets, containing a number of various excipients. PTX-loaded proliposome formulations in solid dosage form are more stable than traditional liposome suspensions (17). Nebulization time of liposomes generated from F3 Micro proliposome tablet formulations (5 ml) was determined (77,78) using Vibrating mesh and Ultrasonic nebulizers. Nebulization time using Vibrating mesh (15.73 ± 1.23 min) for F3 tablets was significantly higher (*p < 0.05*) when compared to the nebulization time of Ultrasonic nebulizer (8.75 ± 0.86 min) (Table VI). The presence of phospholipid and carbohydrate carrier may enhance the viscosity of liposome suspension (78,79), and this may supress the low energy of atomisation employed by Vibrating mesh nebulizer (80). Furthermore, the elevated nebulization time may be associated with liposome vesicle size i.e. large vesicles may cause blockage of mesh aperture, resulting in rupture of liposomes as well as lengthen nebulization time (Table VI). Ultrasonic nebulizer operate based on the movement of piezoelectric crystals and therefore are not affected by liposome vesicle size, consequently this apparatus offered shorter nebulization time (Tables VI and VII). Shorter nebulization times achieved by ultrasonic nebulizers when compared to Vibrating mesh nebulizers are in accordance with the previous findings (81–83).

**Table VI:** PTX-Loaded F3 Micro Proliposome Tablets Analysis Using Vibrating Mesh (i.e. Omron) and Ultrasonic Nebulizer for Nebulization Time (min), Sputtering Time (min), Mass Output (%), and Output Rate (mg/min). Data are Mean ± SD, *n* = 3

Formulations & nebulization type	Nebulization time (min)	Sputtering time (min)	Mass output (%)	Output rate (mg/min)
Vibrating mesh F3	15.73 ± 1.23	0.28 ± 0.06	98.56 ± 3.75	306.72 ± 7.42
Ultrasonic F3	8.75 ± 0.86	1.27 ± 0.08	84.03 ± 2.49	421.06 ± 7.19

**Table VII:** Nebulization of PTX-Loaded F3 Micro Proliposome Tablet Formulation for Size Analysis Employing TSI in Various Compartments (i.e. Nebulizer Reservoir, Upper Stage and Lower Stage). Data are Mean ± SD, *n* = 3

Formulations & nebulization type	Nebulizer reservoir (Size; μm)	Upper stage (Size; μm)	Lower stage (Size; μm)
Vibrating mesh F3	6.86 ± 0.24	3.79 ± 0.16	2.21 ± 0.12
Ultrasonic F3	5.42 ± 0.55	4.12 ± 0.25	2.36 ± 0.25

Similar to nebulization time, sputtering time exhibited a significant difference (*p < 0.05*) for F3 formulation when Vibrating mesh and Ultrasonic nebulizers were employed (Table VI). Sputtering time identified for Vibrating mesh nebulizer was 0.28 ± 0.06 min as compared to 1.27 ± 0.08 min for Ultrasonic nebulizer. The viscosity of F3 tablet formulation did not elevate the sputtering time for Vibrating mesh nebulizer, this may be attributed to Omron’s Vibrating mesh; which may took longer nebulization time but slowly pushed most of the formulation from the nebulizer reservoir. Whereas, nebulization in the Ultrasonic nebulizer is a result of fragmentation of capillary waves, thus the viscosity of liposome suspensions may supress the atomisation process (84). Therefore, a low volume in the nebulizer reservoir may increase the difficulty to form complete waves, lengthening the sputtering time. These results are in agreement with the previous study conducted by Steckel and Eskandar (77).

In summary, the Ultrasonic nebulizer was identified as a superior nebulizer for delivery of aerosol, due to its associated short nebulization time. Whilst the sputtering time was elevated, it did not impact upon complete nebulization time (nebulization plus sputtering time) i.e. an average of 10 min (Ultrasonic nebulizer) then the complete nebulization time of circa 16 min (Vibrating mesh nebulizer).

#### Mass Output and Output Rate of Liposome Suspension

Whilst of complete nebulization of formulations was achieved, this is not a guarantee of complete (100%) mass output, as remnants of the formulation remain in the reservoir, despite nebulization (referred to as dead volume) (85). Upon investigation, a significant difference (*p < 0.05*) in mass output was identified between Vibrating mesh and Ultrasonic nebulizer (Table VI). In spite of the longer nebulization time elicited by the Vibrating mesh nebulizer, its mass output values were high 98.56 ± 3.75%, when compared to the Ultrasonic nebulizer i.e. 84.03 ± 2.49% (Table VI). High mass out via the Vibrating mesh nebulizer was also confirmed by previous investigators (79,86), which may be associated with the lower residual volume in the Vibrating mesh nebulizer (87,88) when compared to Ultrasonic nebulizers.

Output rate exhibited significant differences (*p < 0.05*) between Omron and Ultrasonic nebulizer, as it is time dependent characterization. The output rate for Vibrating mesh nebulizer was found to be 306.72 ± 7.42 mg/min when compared to the 421.06 ± 7.19 mg/min of the Ultrasonic nebulizer (Table VI). Liposome suspension viscosity may also affect the slower rate of nebulization. This higher viscosity resulted in the partial blockage of apertures of the mesh, also due to the large size of liposome vesicles during nebulization. These phenomena may explain the lower output rate (and longer nebulization time) of Vibrating mesh nebulizer. Comparatively, a shorter nebulization time was observed for the Ultrasonic nebulizer, suggesting low impact of the suspension viscosity and thus exhibiting a higher output rate (Table VI).

Overall, the Ultrasonic nebulizer was found to be a more appropriate nebulizer for the aerosolization of liposome suspensions prepared from F3 Micro proliposome tablet formulations, which demonstrated good mass output and high output rate. Contrastingly, the Vibrating mesh nebulizer exhibited high mass output with an elevated nebulization time, which impacted upon the output rate of liposome suspension via aerosolization.

#### Size and PDI Analysis Post Nebulization

Liposomes generated from Micro proliposome tablets F3 formulation were delivered via Vibrating mesh and Ultrasonic nebulizer (Table VII). Nebulizer type and TSI compartments (including nebulizer reservoir, upper and lower stages) impacted upon the measured size of vesicles using size analysis (Table VII) (supplementary data (Fig. f and g)).

Regardless of nebulizer type, size of liposomes which remained in the nebulizer reservoirs were significantly larger (*p < 0.05*) than vesicles delivered to the TSI stages. Liposomes nebulized to the lower stage via TSI using a Vibrating mesh nebulizer exhibited smaller size (*p < 0.05*) than liposomes delivered to the upper stage of impinge (smaller liposomes were incorporated in smaller aerosol droplets) and when compared to vesicles which remained in the nebulizer reservoir (Table VII). These results were analogous to the size of liposomes obtained by the Ultrasonic nebulizer (Table VII). This “size fractionation” of liposomes amongst various compartments of the TSI was also reported when liposomes were delivered via Ultrasonic nebulizer (89), and Vibrating mesh nebulizers (79,90). For both Vibrating mesh and Ultrasonic nebulizers, smaller sized liposomes (than the original liposome vesicles in suspension) were deposited in the lower stage of the TSI (Tables II and VII). Particle size less than 5 μm are considered as respirable fraction, indicating their deposition in the lower respiratory tract (33). PTX-loaded F3 Micro proliposome tablet formulations (Table VII), where were reduced by the shear force of piezoelectric crystal employed by both Vibrating mesh and Ultrasonic nebulizers. Piezoelectric crystal in the Vibrating mesh nebulizer is attached to the transducer horn, which transmit vibrations to the perforated plate (containing 6000 tapered holes with an internal diameter of each hole of 3 μm). Liposome formulations were passed through perforated plate to generate aerosols, which automatically reduce particle size. Whereas, Ultrasonic nebulizer utilize baffle in addition to high frequency vibration by piezoelectric crystal, hence reduce particle size for aerosolization. Similar findings were also reported in various studies (78,91,92). Therefore, these nebulizer generate aerosols with a smaller particles and hence enabled their deposition in the upper and lower stages of TSI.

Overall, significant differences in terms of size was observed for liposomes present in different compartments post nebulization. This difference in size was insignificant in terms of nebulizer type.

### Cell Viability/Cytotoxicity Studies

**Fig. 3:**
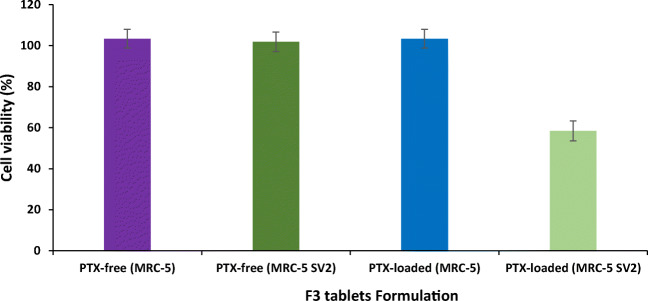
Viability of MRC-5 and MRC-5 SV2 cell lines tested with transfersome generated from proliposome tablet F3 formulation using both PTX-free and PTX-loaded in black, flat-bottomed 96-well plates. Data are mean ± SD, *n* = 4.

In order to find a rapid and simple cell viability study, cytotoxicity of PTX-loaded in proliposome tablets formulation (i.e. F3) against normal MRC-5 and cancerous MRC-5 SV2 cell lines was determined using Alamar Blue assay. The effect of F3 formulation on the viability of MRC-5 and MRC-5 SV2 cells was designed for 24 h (Fig. 3). Employing PTX-free formulations, the cell viability of both cell lines was unaffected (*p* > 0.05), indicating that the manufacture process and formulations parameters of proliposomes (i.e. PTX-free F3 on MRC-5 and MRC-5 SV2) had no effect on the cytotoxicity. Correspondingly, PTX-loaded F3 tablet formulation was safe in MRC-5 cell lines (Fig. 3) (with a PTX concentration of 10% formulations). On the other hand, using similar concentration of PTX was noted to be significantly (*p* < 0.05) toxic to MRC-5 SV2 cell line, exhibiting 58% cell viability. Thus, it was found that PTX-loaded F3 tablets formulation was safe in normal cell lines but significantly toxic to cancer cell lines (Fig. 3).

## Conclusions

This research aimed and proved that Micro or Nano proliposome tablets could be effectively used to deliver PTX loaded into liposomes into the TSI via Vibrating mesh (i.e. Omron) and Ultrasonic nebulizers. From the initial characterization (i.e. angle of repose, entrapment efficiency and size analysis) LMH-based proliposome powder formulations (i.e. F3, F6 and F9) were found better especially in 1:25 *w*/w ratios (regardless of phospholipid type). These three Micro formulations were also probe sonicated in order to reduce the size to Nano liposome, followed by spray drying (i.e. F3SDN, F6SDN, F9SDN and F24SDN). Nano proliposome powder formulations showed good production yield with *poor* to *very poor* compressibility index values. However, among Micro and Nano proliposome tablets formulations (F3, F6, F9, F3SDN, F6SDN and F9SDN), formulation F3 exhibited uniform weight uniformity, high crushing strength, good tablet thickness and short disintegration time. Moreover, nebulization of liposome generated from PTX-loaded Micro proliposome tablets (F3 formulation) demonstrated a better aerosolization characteristics via Ultrasonic nebulizer as compared to Vibrating mesh nebulizer in terms of short nebulization time and high output rate. Thus, it was identified that PTX-loaded Micro proliposome tablets manufactured using LMH as a carrier in a 1:25 w/w lipid phase to carrier ratio are appropriate for the delivery of hydrophobic drug. Cytotoxicity studies demonstrated that proliposome tablet formulations F3 was entirely safe in normal cell lines while toxic to cancer cell lines. This is the first study that demonstrated the possibility of manufacturing anticancer proliposome tablets that can disintegrate in aqueous medium and generate inhalable anticancer liposomes via nebulization. Furthermore, evidence on the cytotoxicity of the anticancer formulation on the cancerous cell were also provided identifying its effectiveness.

## Electronic Supplementary Material

ESM 1 (DOCX 3403 kb)

## Abbreviation

AOR: Angle of repose
DPMC: Dimyristoly phosphatidylcholine
F: Formulation
FD: Freeze dried
FDM: Freeze dried Micro
FDN: Freeze dried Nano
HSPC: Hydrogenated soya phosphatidylcholine
LMH: Lactose monohydrate
MCC: Microcrystalline cellulose
M: Micro
N: Nano
PS: Probe sonication
PTX: Paclitaxel
SEM: Scanning electron microscope
SD: Spray dried
SDM: Spray dried Micro
SDN: Spray dried Nano
SPC: Soya phosphatidylcholine
TEM: Transmission electron microscope
TSI: Two Stage Impinge
PDI: Polydispersity index

## References

[1] Rowinsky EK, Donehower RC (1995). Drug therapy: Paclitaxel (taxol). N Engl J Med.

[2] Panchagnula R (1998). Pharmaceutical aspects of paclitaxel. Int J Pharm.

[3] Yoshizawa Y, Ogawara KI, Kimura T, Higaki K (2014). A novel approach to overcome multidrug resistance: Utilization of P-gp mediated efflux of paclitaxel to attack neighboring vascular endothelial cells in tumors. Eur J Pharm Sci.

[4] Sharma US, Balasubramanian SV, Straubinger RM (1995). Pharmaceutical and physical properties of paclitaxel (Taxol) complexes with cyclodextrins. J Pharm Sci.

[5] Singla AK, Garg A, Aggarwal D (2002). Paclitaxel and its formulations. Int J Pharm.

[6] Garber K (2004). Improved paclitaxel formulation hints at new chemotherapy approach. J Natl Cancer Inst.

[7] Gala RP, Khan I, Elhissi AM, Alhnan MA (2015). A comprehensive production method of self-cryoprotected nano-liposome powders. Int J Pharm.

[8] Karlsson MO, Molnar V, Freijs A, Nygren P, Bergh J, Larsson R (1999). Pharmacokinetic models for the saturable distribution of paclitaxel. Drug Metab Dispos.

[9] Gianni L, Kearns CM, Giani A, Capri G, Vigano L, Lacatelli A (1995). Nonlinear pharmacokinetics and metabolism of paclitaxel and its pharmacokinetic/pharmacodynamic relationships in humans. J Clin Oncol.

[10] Lukyanov AN, Torchilin VP (2004). Micelles from lipid derivatives of water-soluble polymers as delivery systems for poorly soluble drugs. Adv Drug Deliv Rev.

[11] Videira M, Almeida AJ, Fabra A (2012). Preclinical evaluation of a pulmonary delivered paclitaxel-loaded lipid nanocarrier antitumor effect. Nanomedicine.

[12] Khan I, Apostolou M, Bnyan R, Houacine C, Elhissi A, Yousaf SS (2020). Paclitaxel-loaded micro or nano transfersome formulation into novel tablets for pulmonary drug delivery via nebulization. Int J Pharm.

[13] Joshi N, Shirsath N, Singh A, Joshi KS, Banerjee R (2014). Endogenous lung surfactant inspired pH responsive nanovesicle aerosols: Pulmonary compatible and site-specific drug delivery in lung metastases. Sci Rep.

[14] Khan I, Yousaf S, Subramanian S, Alhnan MA, Ahmed W, Elhissi A (2018). Proliposome Powders for the Generation of Liposomes: the Influence of Carbohydrate Carrier and Separation Conditions on Crystallinity and Entrapment of a Model Antiasthma Steroid. AAPS PharmSciTech.

[15] Crosasso P, Ceruti M, Brusa P, Arpicco S, Dosio F, Cattel L (2000). Preparation, characterization and properties of sterically stabilized paclitaxel-containing liposomes. J Control Release.

[16] Koudelka S, Turanek J (2012). Liposomal paclitaxel formulations. Journal of controlled release : official journal of the Controlled Release Society.

[17] Khan I, Yousaf S, Subramanian S, Albed Alhnan M, Ahmed W, Elhissi A (2018). Proliposome tablets manufactured using a slurry-driven lipid-enriched powders: Development, characterization and stability evaluation. Int J Pharm.

[18] Najlah M, Jain M, Wan K-W, Ahmed W, Albed Alhnan M, Phoenix DA (2018). Ethanol-based proliposome delivery systems of paclitaxel for in vitro application against brain cancer cells. Journal of liposome research.

[19] Koudelka Š, Turánek-Knötigová P, MaŠek J, Korvasová Z, Škrabalová M, Plocková J (2010). Liposomes with high encapsulation capacity for paclitaxel: Preparation, characterisation and in vivo anticancer effect**Štěpán Koudelka and Pavlína Turánek-Knötigová contributed equally to this work. J Pharm Sci.

[20] Wang X, Zheng H, Zhu Z, Wei Y, Chen L (2010). Clinical Pharmacokinetics of Paclitaxel Liposome with a New Route of Administration in Human Based on the Analysis with Ultra Performance Liquid Chromatography. J Pharm Sci.

[21] Strieth S, Nussbaum CF, Eichhorn ME, Fuhrmann M, Teifel M, Michaelis U (2008). Tumor-selective vessel occlusions by platelets after vascular targeting chemotherapy using paclitaxel encapsulated in cationic liposomes. Int J Cancer.

[22] Wang X, Zhou J, Wang Y, Zhu Z, Lu Y, Wei Y, *et al*. A phase I clinical and pharmacokinetic study of paclitaxel liposome infused in non-small cell lung cancer patients with malignant pleural effusions. European journal of cancer (Oxford, England : 1990). 2010;46(8):1474–80. 10.1016/j.ejca.2010.02.002 20207133

[23] Hong S-S, Choi JY, Kim JO, Lee M-K, Kim SH, Lim S-J (2016). Development of paclitaxel-loaded liposomal nanocarrier stabilized by triglyceride incorporation. Int J Nanomedicine.

[24] Allen TM (1994). Long-circulating (sterically stabilized) liposomes for targeted drug delivery. Trends Pharmacol Sci.

[25] Colbern GT, Hiller AJ, Musterer RS, Pegg E, Henderson IC, Working PK (1999). Significant Increase in Antitumor Potency of Doxorubicin Hc1 by its Encapsulation in Pegylated Liposomes. Journal of liposome research.

[26] Newman MS, Colbern GT, Working PK, Engbers C, Amantea MA (1999). Comparative pharmacokinetics, tissue distribution, and therapeutic effectiveness of cisplatin encapsulated in long-circulating, pegylated liposomes (SPI-077) in tumor-bearing mice. Cancer Chemother Pharmacol.

[27] Milla P, Dosio F, Cattel L (2012). PEGylation of proteins and liposomes: a powerful and flexible strategy to improve the drug delivery. Curr Drug Metab.

[28] Koudelka Š, Turánek J (2012). Liposomal paclitaxel formulations. J Control Release.

[29] Fauvel M, Farrugia C, Tsapis N, Gueutin C, Cabaret O, Bories C (2012). Aerosolized liposomal amphotericin B: Prediction of lung deposition, in vitro uptake and cytotoxicity. Int J Pharm.

[30] Rudokas M, Najlah M, Alhnan MA, Elhissi A. liposome delivery systems for inhalation: a critical review highlighting formulation issues and anticancer applications. Med Princ Pract. 2016;25(suppl 2)(Suppl. 2):60–72. 10.1159/000445116 PMC5588529 26938856

[31] Hunt CA, Tsang S (1981). α-Tocopherol retards autoxidation and prolongs the shelf-life of liposomes. Int J Pharm.

[32] Khan I, Elhissi A, Shah M, Alhnan MA, Waqar A. Liposome-based carrier systems and devices used for pulmonary drug delivery. In: Davim JP, editor. Biomaterial and medical tribology research and development. 1. Cambridge, UK: Woodhead Publishing Limited, pp:395–443; 2013. p. 395–442.

[33] Khan I, Yousaf S, Alhnan MA, Ahmed W, Elhissi A, Jackson MJ. Design characteristics of inhaler devices used for pulmonary delivery of medical aerosols. In: Ahmed W, Jackson MJ, editors. Surgical Tools and Medical Devices. Cham: Springer International Publishing, pp: 573–591; 2016. p. 573–91.

[34] Wong M, Thompson TE (1982). Aggregation of dipalmitoylphosphatidylcholine vesicles. Biochemistry.

[35] Elhissi AM, Ahmed W, Taylor KM (2012). Laser diffraction and electron microscopy studies on inhalable liposomes generated from particulate-based proliposomes within a medical nebulizer. J Nanosci Nanotechnol.

[36] Khan I, Yousaf S, Subramanian S, Korale O, Alhnan MA, Ahmed W (2015). Proliposome powders prepared using a slurry method for the generation of beclometasone dipropionate liposomes. Int J Pharm.

[37] Payne NI, Timmins P, Ambrose CV, Ward MD, Ridgway F (1986). Proliposomes: a novel solution to an old problem. J Pharm Sci.

[38] Subramanian S, Khan I, Korale O, Alhnan MA, Ahmed W, Najlah M (2016). A simple approach to predict the stability of phospholipid vesicles to nebulization without performing aerosolization studies. Int J Pharm.

[39] Rubin BK, Fink JB (2005). Optimizing aerosol delivery by pressurized metered-dose inhalers. Respir Care.

[40] EPA. Particulate matter USA: Environmental Protection Agency; 2013 [Available from: http://www.epa.gov/airscience/air-particulatematter.htm.

[41] Saker AA, Alanazi FK, Felton LA (2013). Oral solid dosage forms. Remington - Essentials of Pharmaceutics.

[42] Sastry SV, Nyshadham JR, Fix JA (2000). Recent technological advances in oral drug delivery - a review. Pharm Sci Technolo Today.

[43] Jivraj II, Martini LG, Thomson CM (2000). An overview of the different excipients useful for the direct compression of tablets. Pharm Sci Technolo Today.

[44] USP. Powder Flow. Angle of repose, compressability index and hausner ratio. Baltimore, USA: United Book Press; 2015. p. 1326–35.

[45] BP. Powder Flow. Bulk density and tapped density of powder. London, UK: Stationary Office on behalf of MHRA; 2016. p. A526-A47.

[46] BP. Powder Flow. Angle of repose. London, UK: Stationary Office on behalf of MHRA; 2016. p. A526-A47.

[47] Carr R (1965). Evaluating flow properties of solids. Chem Eng.

[48] Maherani B, Arab-tehrany E, Kheirolomoom A, Reshetov V, Stebe MJ, Linder M (2012). Optimization and characterization of liposome formulation by mixture design. Analyst.

[49] Hashtjin AM, Abbasi S (2015). Optimization of ultrasonic emulsification conditions for the production of orange peel essential oil nanoemulsions. J Food Sci Technol.

[50] Kaboorani A, Riedl B. Mechanical performance of polyvinyl acetate (PVA)-based biocomposites. In: Misra M, Pandey J, Mohanty A, editors. Biocomposites: Design and Mechanical Performance. 1. Cambridge, UK: Woodhead Publisher; 2015. p. 347–64.

[51] Harikarnpakdee S, Lipipun V, Sutanthavibul N, Ritthidej GC (2006). Spray-dried mucoadhesive microspheres: Preparation and transport through nasal cell monolayer. AAPS PharmSciTech.

[52] BP. Uniformity of Weight (Mass). London, UK: Stationary Office on behalf of MHRA; 2016. p. A376-A80.

[53] Lachman L, Lieberman HA, Kanig JL. The Theory and Practice of Industrial Pharmacy. 3 ed. 3, editor: Lea & Febiger, Philadelphia, 296–300; 1986.

[54] BP. Disintegration. Disintegration of tablets and capsules. London, UK: Stationary Office on behalf of MHRA; 2016. p. A352-A5.

[55] Hallworth GW, Westmoreland DG (1987). The twin impinger: a simple device for assessing the delivery of drugs from metered dose pressurized aerosol inhalers. J Pharm Pharmacol.

[56] Elhissi AM, Brar J, Najlah M, Roberts SA, Faheem A, Taylor KM (2013). An ethanol-based proliposome technology for enhanced delivery and improved “respirability” of antiasthma aerosols generated using a micropump vibrating-mesh nebulizer. Journal of Pharmaceutical Technology, Research and Management.

[57] McConville JT, Patel N, Ditchburn N, Tobyn MJ, Staniforth JN, Woodcock P (2000). Use of a novel modified TSI for the evaluation of controlled-release aerosol formulations. I Drug Development and Industrial Pharmacy.

[58] Aulton ME, Taylor KMG. Tablets and Compaction. In: Aulton ME, Taylor KMG, editors. Aulton's Pharmaceutics: The Design and Manufacture of Medicines. 5. 5th ed. London, UK: Elsevier Health Sciences, pp: 520–530; 2018. p. 520–30.

[59] Elhissi AMA, Ahmed W, McCarthy D, Taylor KMG (2011). A study of size, microscopic morphology, and dispersion mechanism of structures generated on hydration of proliposomes. J Dispers Sci Technol.

[60] Aulton ME, Taylor KMG. Powder Flow. In: Aulton ME, Taylor KMG, editors. Aulton's Pharmaceutics: The Design and Manufacture of Medicines. 5. 5th ed. London, UK: Elsevier Health Sciences, pp: 189–200; 2018. p. 189–200.

[61] Kan P, Tsao CW, Wang AJ, Su WC, Liang HF (2011). A liposomal formulation able to incorporate a high content of Paclitaxel and exert promising anticancer effect. J Drug Deliv.

[62] Sharma A, Straubinger RM (1994). Novel taxol formulations: preparation and characterization of taxol-containing liposomes. Pharm Res.

[63] You Y-y, Suo X-b, Yue J-j, Xu X, Zhang H. Determination of entrapment efficiency of liposomal paclitaxel by RP-HPLC&#8727. Chinese Journal of Pharmaceutical Analysis. 2017;37(3):535–9.

[64] Rane S, Prabhakar B. Influence of liposome composition on paclitaxel entrapment and pH sensitivity of liposomes. International Journal of PharmTech Research. 2009;1(3):914–7.

[65] Yang T, Cui F-D, Choi M-K, Lin H, Chung S-J, Shim C-K, *et al*. Liposome formulation of paclitaxel with enhanced solubility and stability. Drug Delivery. 2007;14(5):301–8. 10.1080/10717540601098799 17613018

[66] Ashok B, Arleth L, Hjelm RP, Rubinstein I, Onyuksel H (2004). In vitro characterization of PEGylated phospholipid micelles for improved drug solubilization: effects of PEG chain length and PC incorporation. J Pharm Sci.

[67] Campbell RB, Balasubramanian SV, Straubinger RM (2001). Influence of cationic lipids on the stability and membrane properties of paclitaxel-containing liposomes. J Pharm Sci.

[68] He Y, Luo L, Liang S, Long M, Xu H (2019). Influence of probe-sonication process on drug entrapment efficiency of liposomes loaded with a hydrophobic drug. Int J Polym Mater Polym Biomater.

[69] Yeo LK, Chaw CS, Elkordy AA. The effects of hydration parameters and co-surfactants on methylene blue-loaded niosomes prepared by the thin film hydration method. Pharmaceuticals (Basel). 2019;12(2). 10.3390/ph12020046 PMC6631696 30934834

[70] Akbarzadeh A, Rezaei-Sadabady R, Davaran S, Joo SW, Zarghami N, Hanifehpour Y (2013). Liposome: classification, preparation, and applications. Nanoscale Res Lett.

[71] Maury M, Murphy K, Kumar S, Shi L, Lee G (2005). Effects of process variables on the powder yield of spray-dried trehalose on a laboratory spray-dryer. Eur J Pharm Biopharm.

[72] Langrish TAG (2008). Assessing the rate of solid-phase crystallization for lactose: The effect of the difference between material and glass-transition temperatures. Food Res Int.

[73] Murtomaa M, Savolainen M, Christiansen L, Rantanen J, Laine E, Yliruusi J (2004). Static electrification of powders during spray drying. J Electrost.

[74] Bennett FS, Carter PA, Rowley G, Dandiker Y (1999). Modification of electrostatic charge on inhaled carrier lactose particles by addition of fine particles. Drug Dev Ind Pharm.

[75] Goula AM, Adamopoulos KG, Kazakis NA (2004). Influence of spray drying conditions on tomato powder properties. Dry Technol.

[76] Hoppentocht M, Hagedoorn P, Frijlink HW, de Boer AH (2014). Technological and practical challenges of dry powder inhalers and formulations. Adv Drug Deliv Rev.

[77] Steckel H, Eskandar F (2003). Factors affecting aerosol performance during nebulization with jet and ultrasonic nebulizers. Eur J Pharm Sci.

[78] Elhissi AMA, Faizi M, Naji WF, Gill HS, Taylor KMG (2007). Physical stability and aerosol properties of liposomes delivered using an air-jet nebulizer and a novel micropump device with large mesh apertures. Int J Pharm.

[79] Elhissi AM, Karnam KK, Danesh-Azari MR, Gill HS, Taylor KM (2006). Formulations generated from ethanol-based proliposomes for delivery via medical nebulizers. J Pharm Pharmacol.

[80] Ghazanfari T, Elhissi AMA, Ding Z, Taylor KMG (2007). The influence of fluid physicochemical properties on vibrating-mesh nebulization. Int J Pharm.

[81] Mercer TT (1981). Production of therapeutic aerosols; principles and techniques. Chest.

[82] Sterk PJ, Plomp A, van de Vate JF, Quanjer PH (1984). Physical properties of aerosols produced by several jet- and ultrasonic nebulizers. Bull Eur Physiopathol Respir.

[83] Mc Callion ONM, Taylor KMG, Thomas M, Taylor AJ (1996). Nebulisation of monodisperse latex sphere suspensions in air-jet and ultrasonic nebulisers. Int J Pharm.

[84] Boguslaskii Y, Eknadiosyants O (1969). Physical mechanism of the acoustic atomization of a liquid. Soviet Physics - Acoustics.

[85] Clay M, Newman S, Pavia D, Lennard-Jones T (1983). Assessment of jet nebulisers for lung aerosol therapy. Lancet.

[86] Elhissi A, Taylor KMG (2005). Delivery of liposomes generated from pro liposomes using air-jet, ultrasonic and vibrating-mesh nebulisers. J Drug Del SciTechnol.

[87] Dolovich MB, Dhand R (2011). Aerosol drug delivery: developments in device design and clinical use. Lancet.

[88] Vecellio L, Abdelrahim ME, Montharu J, Galle J, Diot P, Dubus J-C (2011). Disposable versus reusable jet nebulizers for cystic fibrosis treatment with tobramycin. J Cyst Fibros.

[89] Leung KKM, Bridges PA, Taylor KMG. The stability of liposomes to ultrasonic nebulisation. Int J Pharm 1996;145(1–2):95–102.

[90] Elhissi A, Gill H, Ahmed W, Taylor K (2011). Vibrating-mesh nebulization of liposomes generated using an ethanol-based proliposome technology. Journal of liposome research.

[91] Elhissi A, Hidayat K, Phoenix DA, Mwesigwa E, Crean S, Ahmed W (2013). Air-jet and vibrating-mesh nebulization of niosomes generated using a particulate-based proniosome technology. Int J Pharm.

[92] Dhand R. Nebulizers that use a vibrating mesh or plate with multiple apertures to generate aerosol. Respir Care. 2002;47(12):1406–16; discussion 16–8. 12467499

