# Supplementary material for: A Facile and Novel Approach to Manufacture Paclitaxel-Loaded Proliposome Tablet Formulations of Micro or Nano Vesicles for Nebulization
Source: Pharm Res. 2020 Jun 2;37(6):116. doi: 10.1007/s11095-020-02840-w (PMC7266847; doi:10.1007/s11095-020-02840-w)
Supplement: Supplementary file 1 — (DOCX 3403 kb) [file 11095_2020_2840_MOESM1_ESM.docx]

**Supplementary Data**


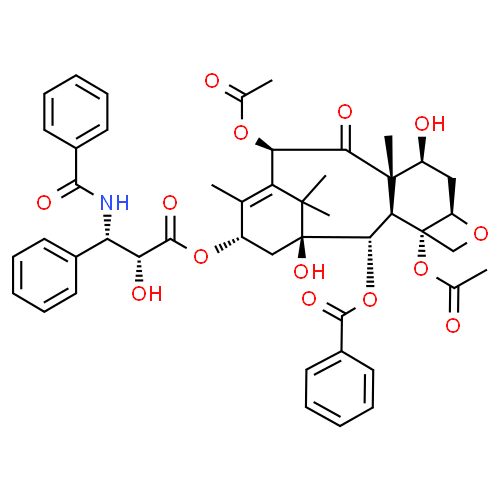


**Figure a**. Chemical structure of paclitaxel using ChemDraw software


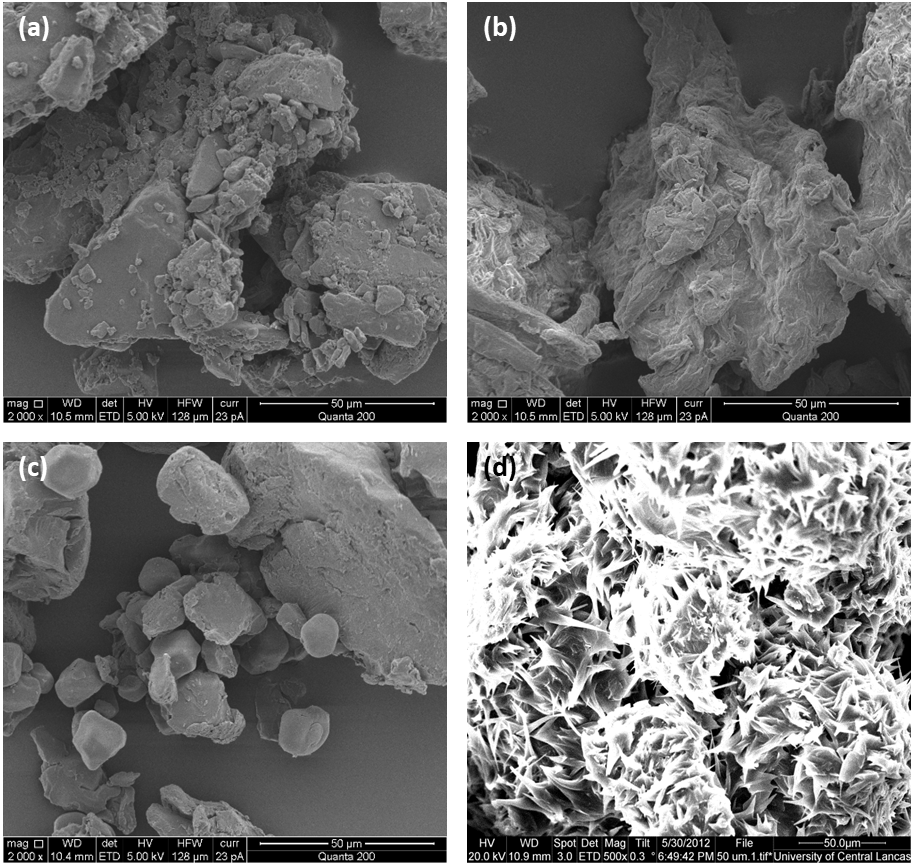


**Figure b**. SEM images of coarse non-porous carbohydrate carriers including; (a) lactose monohydrate, (b) microcrystalline cellulose, (c) Starch, and coarse porous carbohydrate carrier (d) sorbitol. These images clearly distinguish between porous and non-porous coarse carbohydrate carrier partices.


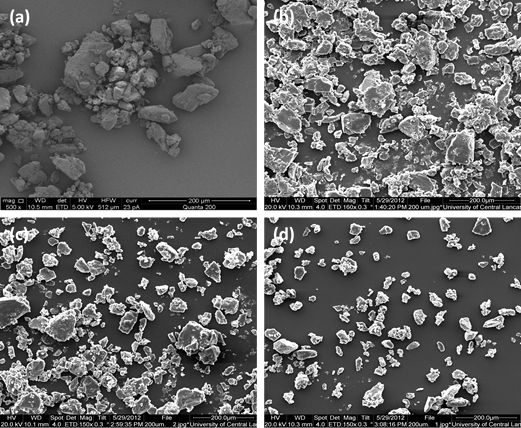


**Figure c**. SEM images of coarse carbohydrate carrier and proliposome powder prepared with different w/w lipid phase to carrier ratios including; (a) coarse lactose monohydrate, (b) proliposmoe with 1:5 w/w, (c) proliposmoe with 1:15 w/w, and (d) proliposmoe with 1:25 w/w. These images are typical of four different experiments


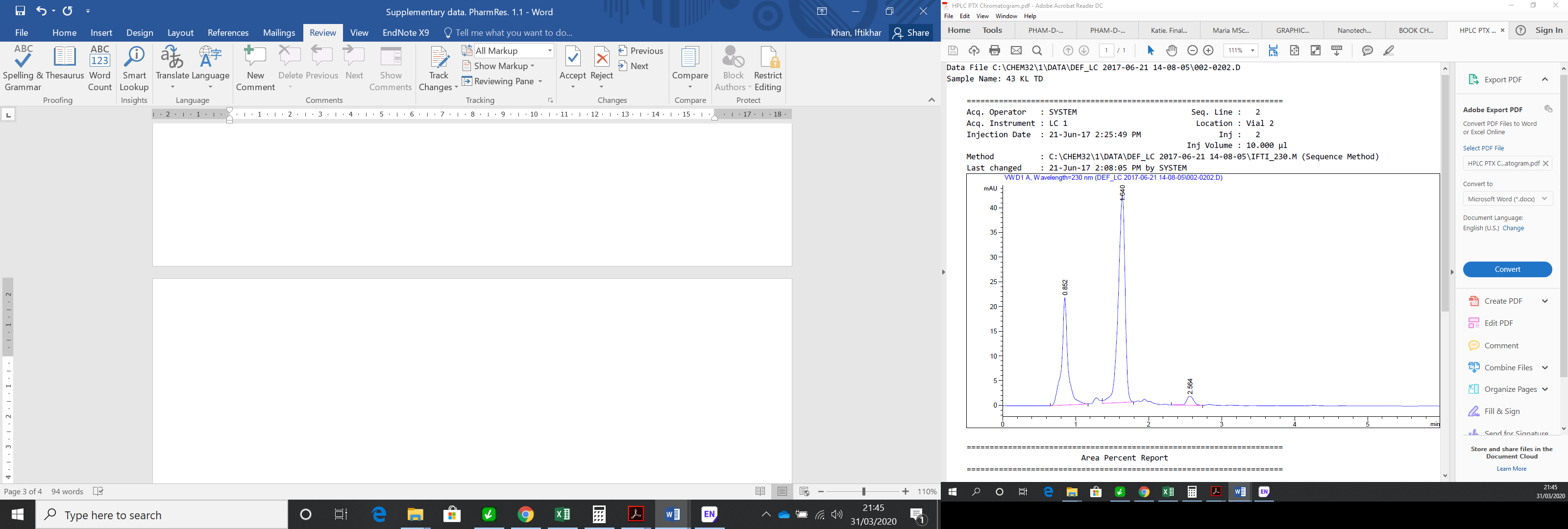


**Figure d.** Chromatogram of PTX via high performance liquid chromatography.


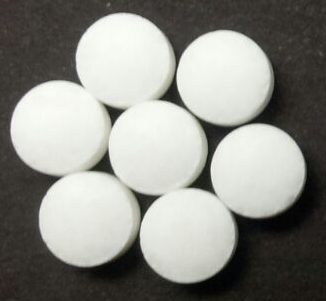


**Figure e.** PTX-loaded proliposome flat tablets (i.e. 10 mm in diameter) compressed from proliposome powder via Stylcam 2000R compaction simulator compression machine.


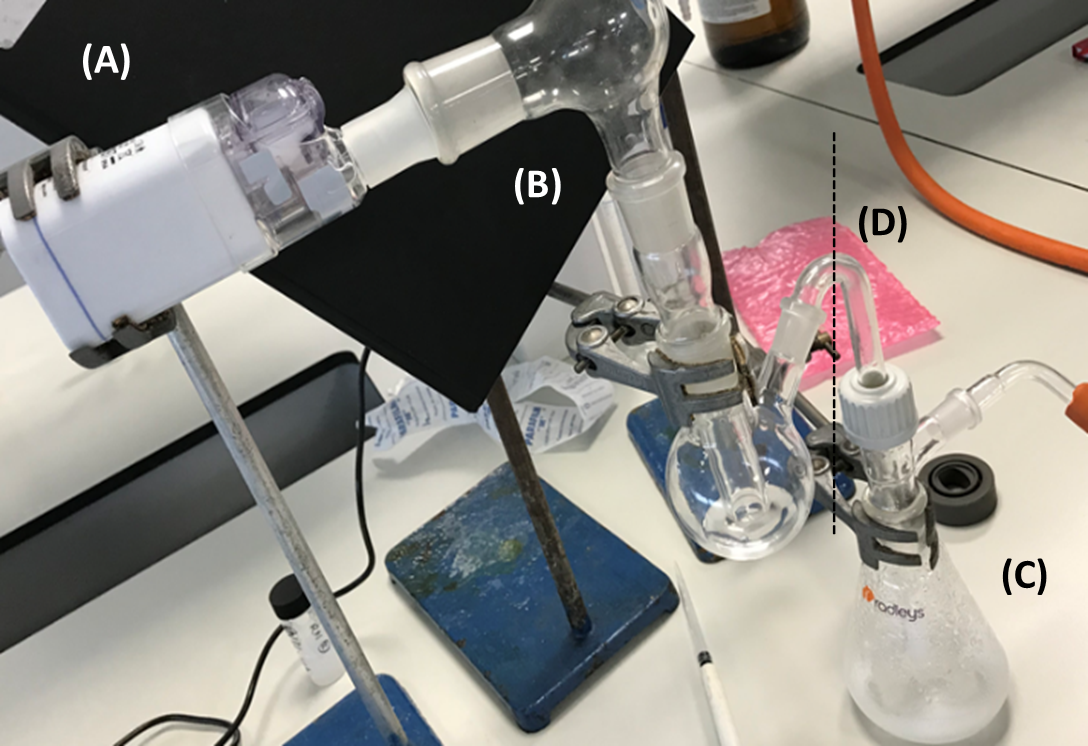


**Figure f.** Representing, (A) Vibrating mesh nebulizer next to the two stage Impinger containing, (B) upper stage and, (C) lower stage and, (D) cut-off diameter between the upper and lower stages for aerosol deposition.


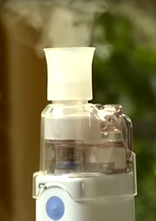


**Figure g.** Liposome suspension aerosolization using Vibrating mesh nebulizer.
